# Chitosan‐Carbon Dot Composite Materials Form a Leaf Surface Barrier to Mitigate the Enrichment and Invasion of Nanoplastics: From Leaf Interface to Systemic Response

**DOI:** 10.1002/advs.75278

**Published:** 2026-04-16

**Authors:** Beibei Zhao, Mei Li, Mengjiao Fan, Chuanhuan Liu, Jie Jiang, Jia Song, Yingzhu Liu

**Affiliations:** ^1^ School of Forestry and Landscape Architecture Anhui Agricultural University Hefei China

**Keywords:** air pollution, carbon dots, chitosa, leafy vegetables, metabolome, polystyrene nanoplastic, transcriptome

## Abstract

To address the potential risk posed by the atmospheric deposition of micro‐nano plastics (M/NPs) to agricultural product safety, this study develops a chitosan‐carbon dot (CS‐CDs) composite suitable for foliar spraying. Its mechanism for alleviating polystyrene nanoplastic (PS) stress is systematically investigated at two growth stages (day 35 and 45) of *Brassica rapa*. Results demonstrate that CS‐CDs form a homogeneous protective film on the leaf surface, which persistently and effectively blocks PS attachment and stomatal intrusion. Concurrently, the CDs component is absorbed by the plant and undergoes systemic translocation, synergistically enhancing plant resistance both externally and internally. By day 45, the fresh and dry weights of *Brassica rapa* increase by 26.17% and 30.77%, respectively. Oxidative damage is effectively mitigated. Photosynthesis has also been improved. Furthermore, the foliar microbial community exhibits a 103.93% increase in the Shannon index and shows enhanced richness of *Pseudomonadota*, *Actinomycetota*, and *Chloroflexota*. Defense pathways including *keratin, cork, and wax biosynthesis* and *phenylpropane metabolism* are also activated. This study elucidates the integrated mechanism by which CS‐CDs alleviate PS stress across physiological, biochemical, and microbial levels, providing a novel strategy for using nanomaterials to reduce M/NPs accumulation and toxicity in plants.

## Graphical abstract

Foliar spraying of CS‐CDs can form a film on the leaves of *Brassica rapa*, effectively reducing the enrichment and absorption of PS in the leaves, while increasing the biomass and nutrient content of the plants. In addition, CS‐CDs can also enrich the interfoliar microbial community and activate the plant's own defense metabolic pathways.

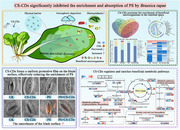

## 1 Introduction

In recent years, the global annual production of plastics has exceeded 400 million tons [1]. Micro‐nanoplastics (M/NPs) formed during environmental degradation are widely present in the atmosphere, causing severe environmental pollution [2]. Among them, nanoplastics (NPs) with a particle size of less than 10 µm can enter the terrestrial ecosystem through atmospheric deposition [2, 3]. Leaves, as the main interface between plants and the atmospheric environment, are highly prone to adsorbing NPs in the atmosphere [1, 3]. Studies have shown that the aggregation of NPs in the atmosphere on plant leaves can block stomata, interfere with gas exchange, and photosynthesis [4]. In addition, these NPs can enter the mesophyll tissue through stomata and cuticle via the epidermal pathway and migrate along the vascular bundle cell wall [2, 3, 5, 6, 7, 8, 9], and then undergo long‐distance transportation within the plant body through the plasmolysis pathway, further triggering the generation of a large amount of reactive oxygen species (ROS), damaging the cell structure and function of plants [5, 10]. For example, Li et al. studied that after NPs enter corn leaves through stomata, it cause oxidative stress in plants, disrupt the structure and function of cell membranes, reduce the content of membrane‐related lipids and gene expression, etc., causing negative impacts [11]. Shi et al. also indicated that after NPs enter the plant body through stomata and cuticle, they are transported in the plant body through the plasmolysis and periplasmic pathways, which not only causes oxidative stress but also changes the microbial community structure and metabolite composition in the inter‐leaf area [5].

Among various types of NPs, polystyrene NPs (PS) are regarded as the most widely distributed and easily absorbed by plants [2, 3, 12]. Studies have shown that PS has been detected in edible crops such as *Lactuca sativa* [13], *Solanum lycopersicum* [5], and *cherry radishes* [14]. However, although previous research has clarified the absorption and stress response mechanisms of plants to PS, there are still relatively limited studies on how to reduce the enrichment and absorption of PS on leaf surfaces in the atmosphere and alleviate the oxidative stress caused by PS to plants [1]. Among various leafy vegetables, *Brassica rapa* is a widely consumed vegetable type rich in minerals and vitamins. Its broad leaves and complex surface structure make it easy to adsorb and accumulate particles in the air (such as M/NPs and dust) [3, 8, 15], which can pose serious threats to human health through the food chain [16]. Moreover, due to its short growth cycle, it has become an ideal model material for studying the impact of NPs in the atmosphere on leafy vegetables [8]. However, research on reducing the absorption of PS by *Brassica rapa* remains limited. Therefore, it is urgently necessary to clarify the physiological and ecological effects of PS in the air on *Brassica rapa* leaves and actively explore effective mitigation strategies.

When seeking strategies to reduce the toxic effects of environmental pollutants on plants, biobased materials and nanotechnology have shown significant potential [17]. Among environmentally friendly materials, such as chitosan (CS) [18] and carbon dots (CDs) [19, 20], have been widely used in enhancing crop stress resistance research. CS is a natural polysaccharide derived from the shells of crustaceans, with excellent biocompatibility, degradability, and film‐forming ability [18, 21, 22]. Previous studies have shown that in alleviating non‐biological stress in crops such as *Zea mays*, *Oryza sativa*, and *Solanum lycopersicum*, the mechanism of CS mainly includes enhancing photosynthesis in plants and activating the antioxidant defense system, etc. [23, 24]. CDs, as a new type of carbon‐based nanomaterial, are highly regarded for their excellent biocompatibility as well as their unique physical and chemical properties [12]. In addition, CDs not only have antioxidant enzyme‐like activity, which can remove reactive oxygen species in the plant body and alleviate oxidative damage, but also participate in stress response regulation as signaling molecules [12, 25, 26]. Although CS and CDs have been extensively studied in terms of alleviating crop stress, the research on their mechanism of action mainly focuses on physiological and biochemical regulation (such as inducing antioxidant enzyme activity, removing ROS, etc.), while studies on their formation of a barrier on the leaf surface to reduce the absorption of NPs by the crops and simultaneously regulate the internal physiological responses of the plants have not been fully carried out.

Based on the unique characteristics of NPs in the atmospheric stress types, this study combined CS and CDs to form a composite material (CS‐CDs) and sprayed it onto the surface of plant leaves to explore its synergistic effect and mechanism in alleviating PS stress. Different from previous studies, CS has transformed from a biological alleviation function to a physical barrier matrix. Its film‐forming property is utilized to improve the smoothness of the leaf surface and form a persistent protective layer, thereby blocking the attachment and invasion of PS. At the same time, CDs, in collaboration with CS to optimize the leaf inter‐microbial community, are absorbed and transported by the leaves to various parts of the plant, exerting their inherent antioxidant activity and photosynthetic promotion function. This further synergistically activates the plant's intrinsic defense pathways, enhancing the overall stress resistance at the system level. Through integrated multi‐omics analysis, this study confirmed that CS‐CDs can combine the physical protection on the leaf surface with the physiological regulation within the plant, effectively improving the plant's resistance to atmospheric NPs stress. This research provides new strategies for reducing crop absorption of atmospheric NPs and reducing the ecological risk of their transfer along the food chain, and also provides a new theoretical basis for the application of multifunctional nanomaterials in agricultural production safety.

## 2 Results and Discussion

### 2.1 Characterization Analysis of CDs and CS‐CDs

**FIGURE 1:**
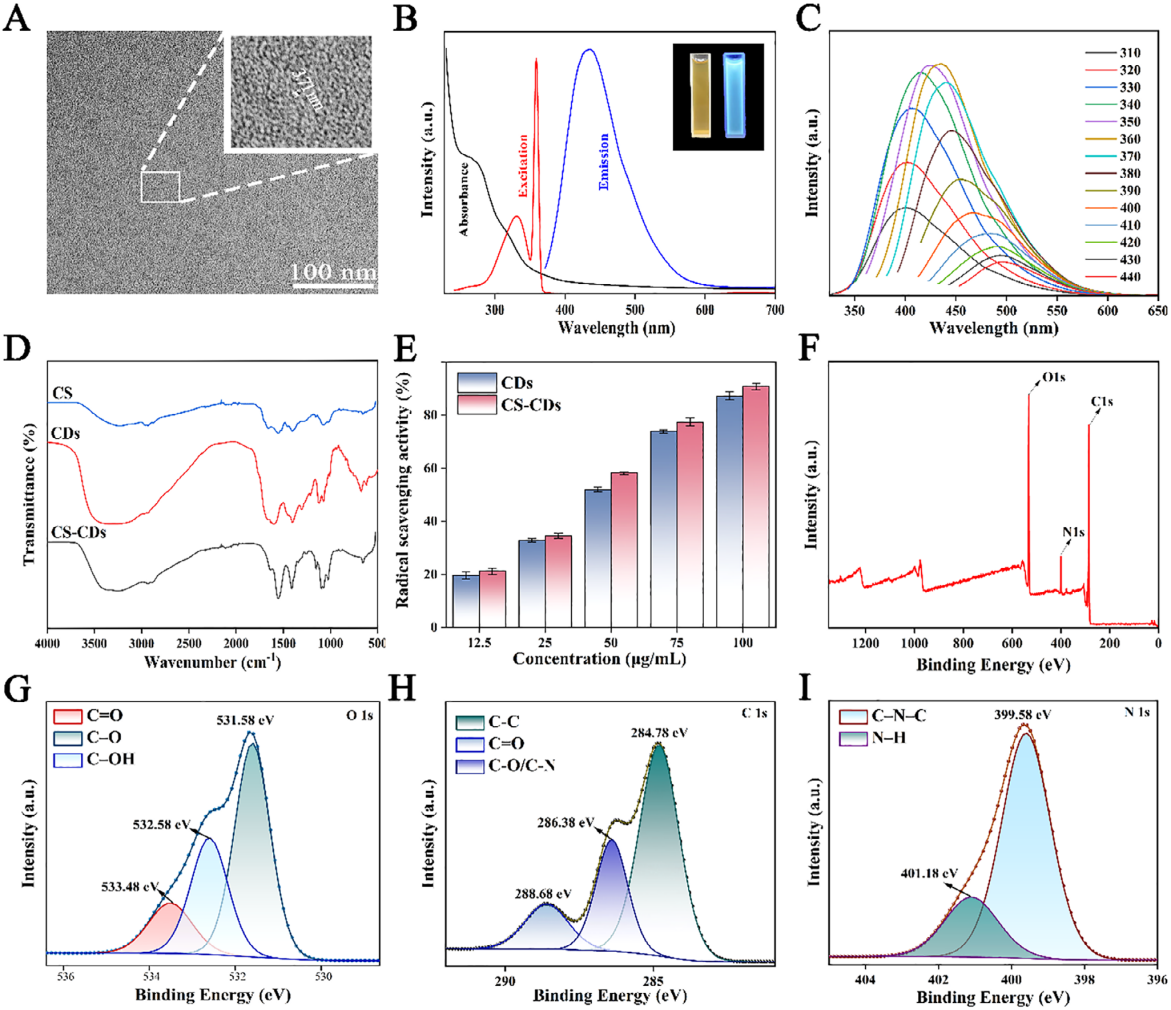
The characterization of CDs, PS, and CS‐CDs. (A) TEM image of CDs, scale bar = 100 nm. (B) UV–vis absorption, fluorescence excitation, and emission spectra (Inset: CDs solution under daylight and 365 nm UV light). (C) Fluorescence emission spectra of CDs solution at different excitation wavelengths (310–440 nm). (D) FTIR spectra of CS, CDs, and CS‐CDs. (E) Radical scavenging activities of CDs and CS‐CDs against DPPH. (F) XPS survey spectrum of CDs and (G–I) high‐resolution O 1s, C 1s, N 1s.

In this study, fluorescent CDs were successfully synthesized by the hydrothermal method using the pods of *Cercis chinensis* as the carbon source. Transmission electron microscopy (TEM) images showed that these CDs were uniformly dispersed without obvious agglomeration (Figure 1A), and the particle size statistics indicated that their sizes were concentrated within the range of 2–5 nm (Figure S1), which was in line with the quantum size classification criteria [12], facilitating absorption and transportation within plant tissues [27]. The UV–vis absorption spectrum shows that the CDs have a distinct absorption band near 290 nm (Figure 1B, black line), which may be related to the π–π* transitions caused by functional groups such as C═O and C─N [28]. Under the excitation of 365 nm UV light, the CDs aqueous solution emits bright blue fluorescence (inset of Figure 1B), demonstrating the typical luminescent behavior of fluorescent nanomaterials [12]. Fluorescence spectroscopy analysis indicated that as the excitation wavelength increased from 310 to 440 nm, the emission peak underwent a systematic redshift (Figure 1C). This might be due to the differences in the size and surface functional groups of CDs, which led to a continuous change in the emission wavelength with the alteration of the excitation light [29, 30]. When excited at 360 nm, the CDs show the strongest emission peak at 436 nm, further verifying its excellent fluorescence performance [12]. Fourier transform infrared spectroscopy (Figure 1D) shows that, compared with CDs, the absorption peak belonging to the O─H/N─H stretching vibration of CS‐CDs at approximately 330 cm^−^^1^ shows a slight broadening and peak shape change, which may be due to the formation of a hydrogen bond network between CS and CDs [31]. At approximately 2920 cm^−^^1^, the absorption peak of CS‐CDs shifted compared with that of CS and CDs, which might be attributed to the tensile vibration of C‐H [21]. Furthermore, within the range of 2300–1950 cm^−^^1^, the spectra of CS‐CDs showed significant fluctuations compared with CDs. It is speculated that this is due to the introduction of CS enriching the group vibration modes in this region. In the carbonyl region, the intensity of the C═O stretching vibration absorption peak of CS‐CDs at approximately 1640 cm^−^^1^ is enhanced compared with that of CDs, which may be attributed to the increase in the stretching vibration absorption intensity of the C═O group in CS‐CDs [21]. The above results indicate that the combination of CS and CDs does not change the basic chemical structure of the polymer chain, but causes displacement, intensity, and morphological changes of multiple characteristic absorption peaks. These phenomena jointly suggest that there is an intermolecular interaction between the two and they show good compatibility. The results of the 2,2‐diphenyl‐1‐picrylhydrazyl (DPPH) radical scavenging experiment showed that both CDs and CS‐CDs exhibited concentration‐dependent antioxidant capacity (Figure 1E). At 100 µg/mL, the clearance rates of CS‐CDs and CDs were 90.82% and 87.23%, respectively. Compared with CDs, the higher free radical scavenging activity of CS‐CDs can be attributed to the synergistic effect between CDs and CS. CDs itself has the ability to scavenge free radicals, and CS also has antioxidant properties [23, 25]. These two may have formed amide bonds through amino and carboxyl groups, optimizing the electron transfer pathway and enhancing the proton donor capacity, thereby synergistically improving the antioxidant performance of CS‐CDs. The X‐ray photoelectron spectroscopy (XPS) spectrum (Figure 1F) shows the characteristic peaks of CDs, located at 531.08 eV (O 1s), 285.08 eV (C 1s), and 400.08 eV (N 1s), indicating that CDs are mainly composed of oxygen, carbon, and nitrogen [12]. The high‐resolution O 1s spectrum can be decomposed into three subpeaks: 531.58 eV (C─O), 532.58 eV (C─O─H), and 533.48 eV (C═O) (Figure 1G). The C 1s spectrum can be fitted into three subpeaks: 284.78 eV (C─C /C═C), 286.38 eV (C─O/C─N), and 288.68 eV (C═O) (Figure 1H). The N 1s spectrum contains two characteristic peaks of 399.58 eV (C─N─C) and 401.18 eV (Figure 1I). This is consistent with the research results reported by Liu and Cheng et al., further confirming the success of the synthesis of CDs in this study [12, 32]. In conclusion, the CDs successfully synthesized in this study have good compatibility with CS. The addition of CDs endows CS‐CDs with more functional groups, thereby enhancing the antioxidant performance of CS‐CDs. The positive synergistic effect exhibited by the combination of CS and CDs provides a solid material foundation for subsequent research on their application in reducing the accumulation and absorption of PS in plant leaves and enhancing the plant's resistance to PS.

### 2.2 The Leaf Surface Film‐Forming Properties of CS‐CDs and the Absorption and Transport of CDs Within Plants

**FIGURE 2:**
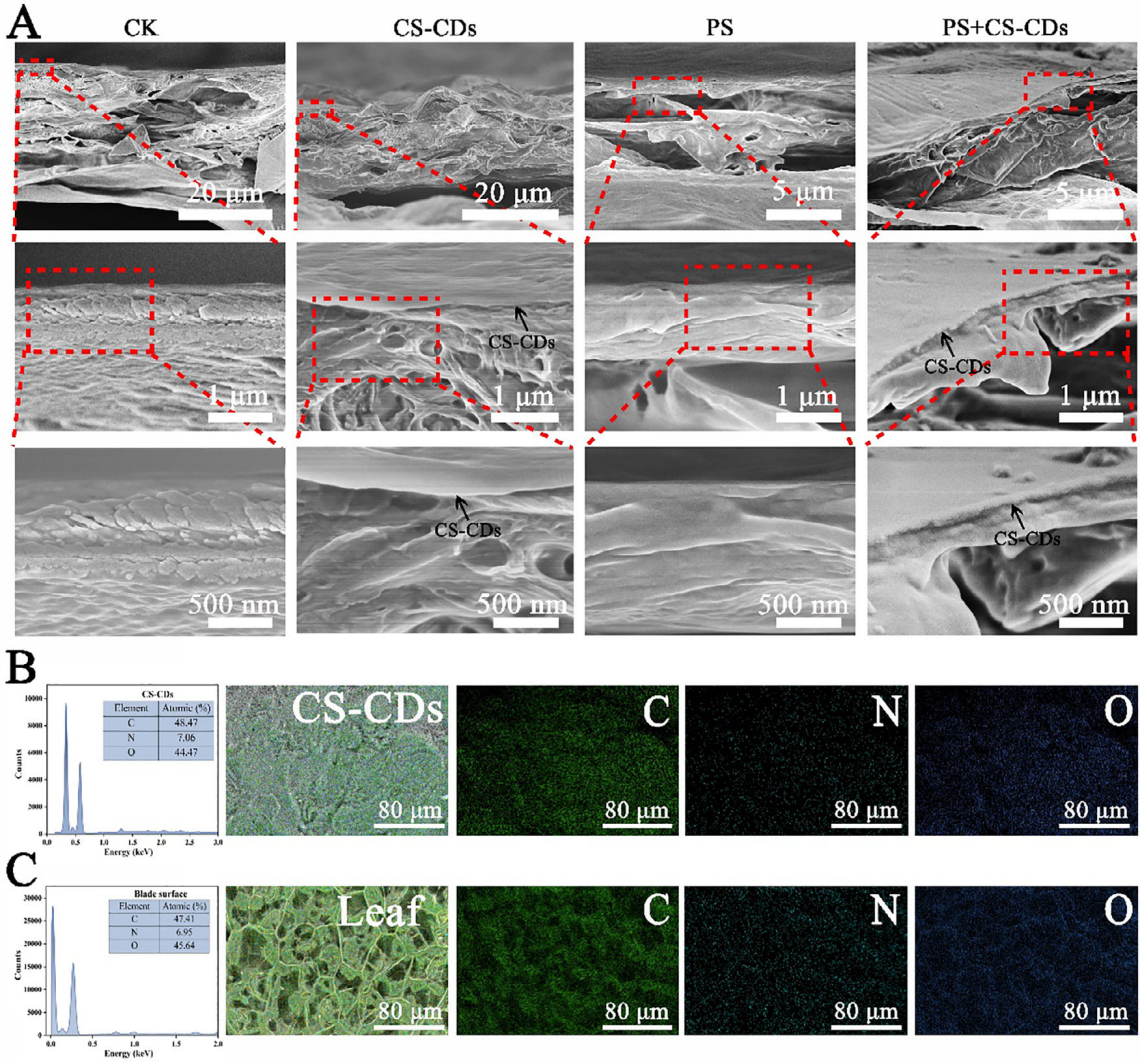
Adhesion of CS‐CDs on *Brassica rapa* leaf surfaces. (A) SEM analysis of cross‐sectioned *Brassica rapa* leaves at 45 days post‐emergence. Scale bar = 20 µm/1 µm/500 nm. (B) SEM‐EDS analysis of CS‐CDs films. Scale bar = 80 µm. (C) SEM‐EDS analysis of the CS‐CDs film on the surface of the leaf.

Scanning electron microscopy (SEM) observations and energy dispersive spectroscopy (EDS) analyses revealed that CS‐CDs formed a uniform and dense protective film on the leaf surface without causing any damage to the leaf structure (Figure 2A–C). This characteristic may be attributed to the excellent biocompatibility of CS and CDs materials [31]. Compared with other similar molecular materials (such as sodium alginate, pectin, starch, etc.), CS contains amino and carboxyl groups, providing ideal sites for the binding with CDs. The two may enhance the structural density and smoothness of the membrane through hydrogen bond interactions (Figure 1G), making the CS‐CDs membrane more flat, smooth, and compact [31]. In addition, CS has the function of inducing plant resistance, which can better maintain the physiological activity of plants [10, 31], and CS has limited solubility in neutral aqueous solutions, and the formed coating is not easily dissolved or washed away by rainwater [33], thus improving its persistence on the leaf surface.

**FIGURE 3:**
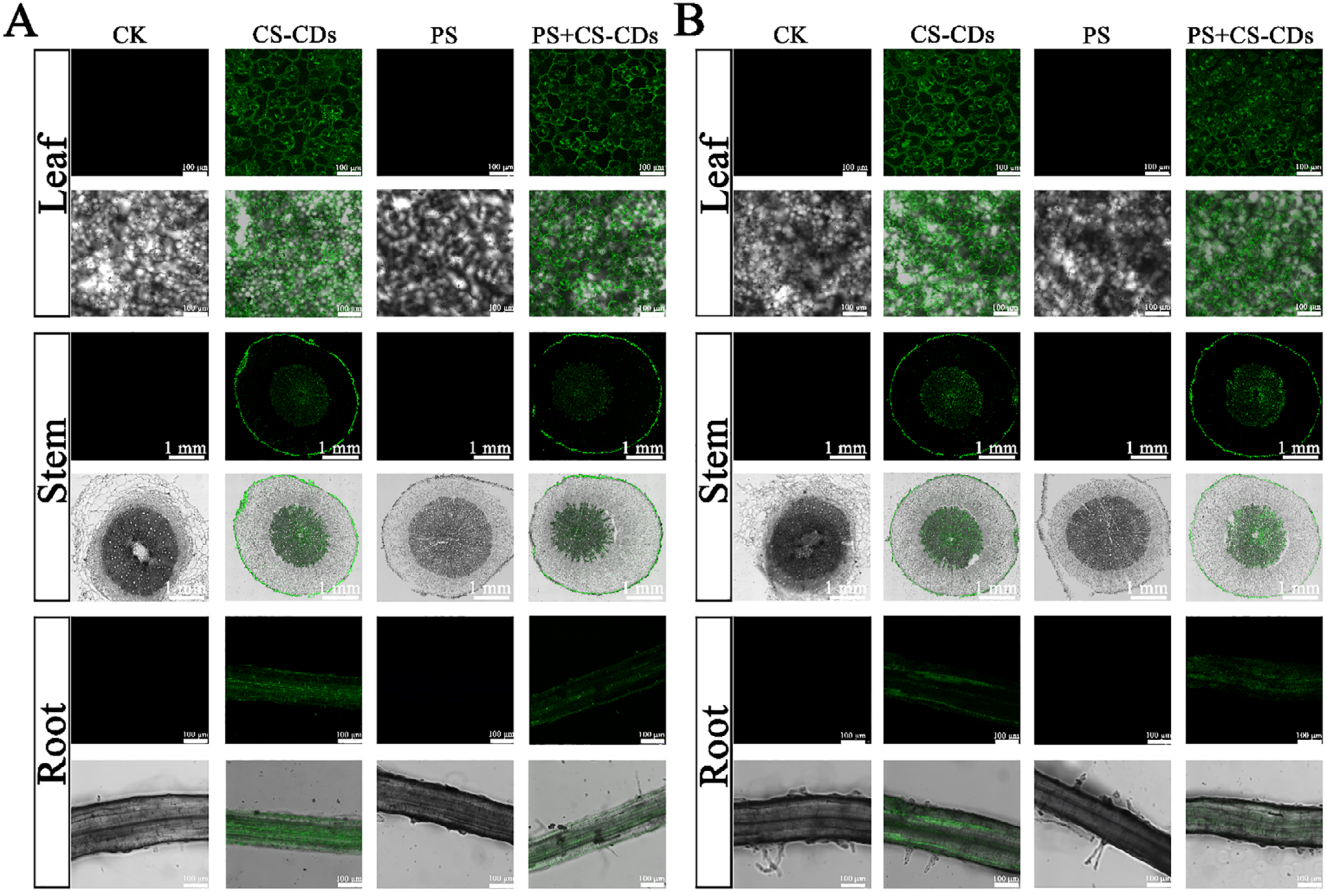
Absorption and transport of CDs within plant tissues. CDs absorption and transport in the leaves, stems, and roots of bok choy at 35 days (A) and 45 days (B) after growth initiation. Scale bar = 100 µm/1 mm.

To investigate whether the CDs loaded on CS‐CDs can enter the plant body, this study utilized the characteristic of CDs emitting green fluorescence at a specific wavelength to conduct systematic fluorescence imaging analysis of the roots, stems, and leaves of *Brassica rapa*. Figure 3A,B shows that on the 35th and 45th days, obvious green fluorescence was observed in the roots, stems and leaves of the CS‐CDs group and the PS+CS‐CDs group of *Brassica rapa*, indicating that CS‐CDs can continuously release CDs, and CDs, with their smaller particle size, can be absorbed by the *Brassica rapa* leaves and transported to the roots through vascular bundles and phloem [27]. In contrast, other carbon‐based materials (such as biochar) are usually at the micrometer level and difficult to enter the plant body for systematic regulation through stomata or cuticle [34], and do not have fluorescence characteristics, making it difficult to observe their distribution in the body. Moreover, CDs have been widely confirmed to have good biocompatibility [12], while other carbon‐based materials may have potential biological toxicity and are not suitable for edible crops. In conclusion, CS and CDs can utilize their own advantages to integrate the dual functions of forming a physical barrier on the leaf surface and systematic regulation in the plant body.

### 2.3 CS‐CDs Synergistically Regulate to Alleviate the Inhibition of PS Stress on the Growth and Photosynthetic Performance of *Brassica rapa*

**FIGURE 4:**
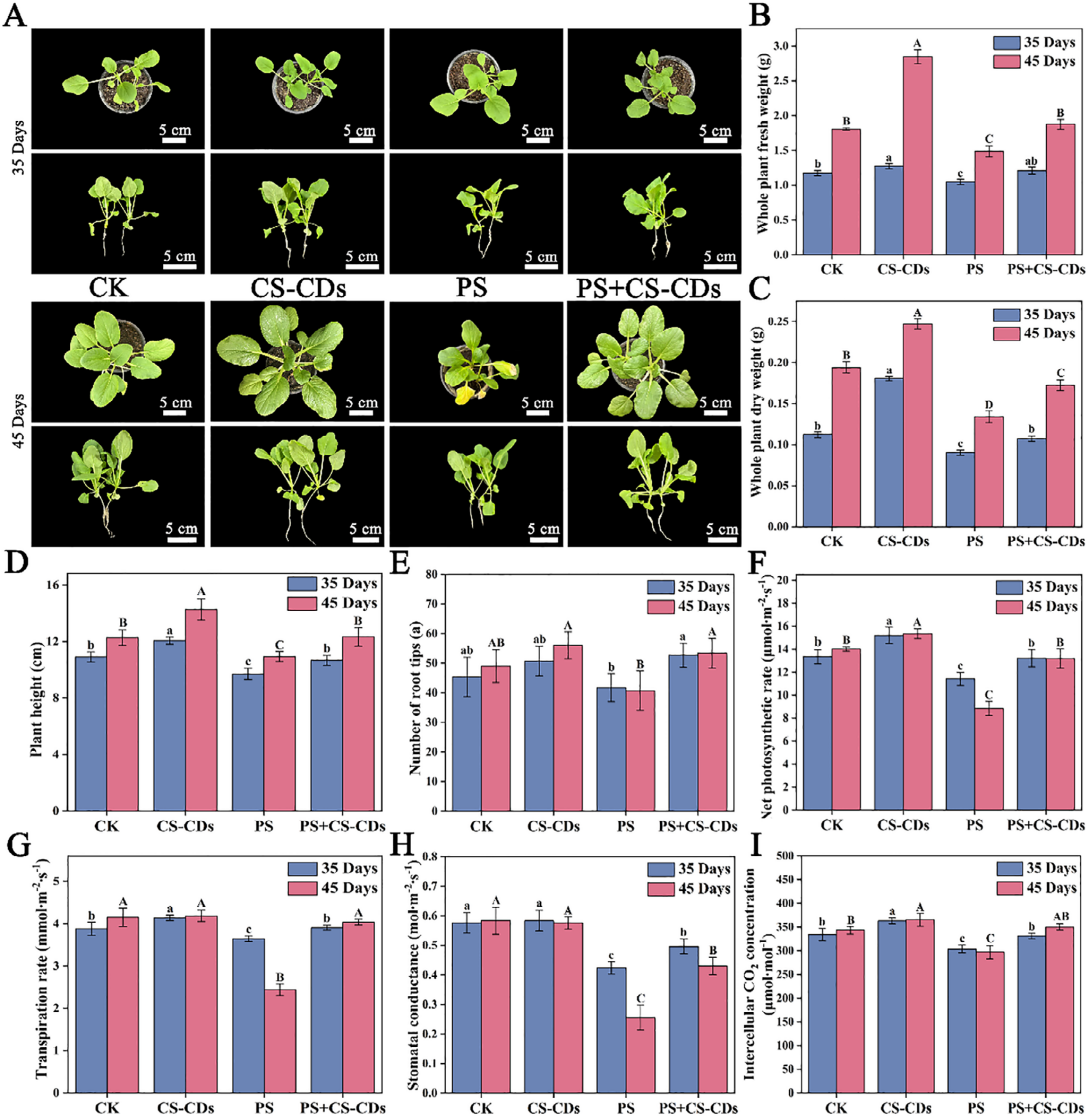
Using CK as the control, the effects of CS‐CDs, PS, and PS+CS‐CDs on the growth and photosynthesis of *Brassica rapa* were investigated. (A) The growth conditions of *Brassica rapa* on the 35th and 45th days. scale bar = 5 cm. (B) Whole plant fresh weight. (C) Whole plant dry weight. (D) Plant length. (E) Number of root tips. (F) Net photosynthetic rate. (G) Transpiration rate. (H) Stomatal conductance. (I) Intercellular CO_2_ concentration. Data are presented as mean ± standard deviation (n = 6). Bars indicated with different letters indicate significantly different values (*p* < 0.05).

This study further evaluated the effects of CS‐CDs and PS on the growth of *Brassica rapa*. Through phenotypic and growth indicators (Figure 4A–F), it was shown that short‐term (on the 35th day) PS exposure only caused a slight inhibition, while long‐term (on the 45th day) stress led to typical stress symptoms, such as yellowing of leaves and weakening of the plants. This phenomenon is consistent with the report by Yan et al. [14]. The CS‐CDs treatment not only significantly promoted plant growth but also effectively alleviated the inhibitory effect caused by PS. At day 45, the various indicators of the PS+CS‐CDs group were respectively 26.17%, 30.77%, 12.81%, and 31.13% higher than those of the PS group. This might be because CS‐CDs effectively alleviate the stress caused by PS, because CS‐CDs are different from traditional fertilizers (such as nitrogen fertilizer, phosphorus fertilizer, and potassium fertilizer). Unlike traditional fertilizers, CS‐CDs do not promote plant growth by directly providing the necessary nutrients to the plants; instead, their main function is to provide protection and regulation.

Studies have shown that M/NPs in the air mainly inhibit plant growth by affecting photosynthesis [2, 35]. For example, Shi et al. demonstrated that high doses of PS would significantly reduce the photosynthetic parameters of *Solanum lycopersicum* leaves [5]. In this study, the same result was also found, which might be because PS would adhere to the leaf surface, and its shading effect would disrupt the normal photosynthesis of plants [5]. Chlorophyll and carotenoids are not only the main pigments in the photosynthetic system for absorbing, transporting, and converting light energy [36], but their content is also an important indicator for measuring the integrity and functional state of the plant's photosynthetic mechanism [9, 37]. Compared with the CK group, the chlorophyll and carotenoid contents in the PS group continued to decrease with the extension of stress time (Figure S2). This might be because PS would inhibit the electron transfer in the light reaction, thereby reducing the photosynthesis of chloroplasts and the content of photosynthetic pigments [38]. The reduction in photosynthetic pigment content would cause damage to the light capture ability of photosystem II and antennae systems, thereby further reducing the energy supply to the plant [38, 39]. The decrease in biomass in Figure 4A–E further supports this result. On the 45th day, the net photosynthetic rate (Pn), transpiration rate (Tr), stomatal conductance (Gs), intercellular carbon dioxide concentration (Ci), chlorophyll and carotenoid contents in the PS+CS‐CDs group were respectively 49.21%, 65.57%, 65.38%, 17.85%, 47.37% and 33.33% higher than those in the PS group (Figure 4F–I, Figure S2, *p *< 0.05). This might be because CS‐CDs reduced the enrichment and invasion of PS on the leaves, and these CDs loaded on CS‐CDs could enter the plant body as electron donors and acceptors, regulating the electron transfer between photosystem I and II, thereby promoting oxygen production, water splitting and the reduction cycle of NADP to NADPH, allowing energy to be transferred to the chloroplasts (Figure 10B) [40]. In addition, CDs can also utilize their photoluminescence properties to convert light energy that plants cannot absorb into absorbable light, thereby improving plant photosynthesis [12, 41]. This effect has also been confirmed in the alleviation of light inhibition in *Chlorella pyrenoidosa* by CDs [40]. The enhancement of photosynthesis and the increase in photosynthetic pigment content indicate that CS‐CDs effectively alleviate the damage to the photosynthetic system caused by PS, providing a necessary energy source for plant growth and development [42].

In conclusion, this study reveals the inhibition of PS on the growth process and photosynthesis of plants, and confirms that applying CS‐CDs on the leaf surface can alleviate PS stress through the synergistic effect of physical barrier and physiological regulation without causing additional stress, providing a feasible strategy for reducing the accumulation of M/NPs in leafy vegetables.

### 2.4 CS‐CDs Can Alleviate the Cell Damage and Oxidative Stress Caused by PS, and at the Same Time Enhance the Activity of Antioxidant Enzymes in *Brassica rapa*

**FIGURE 5:**
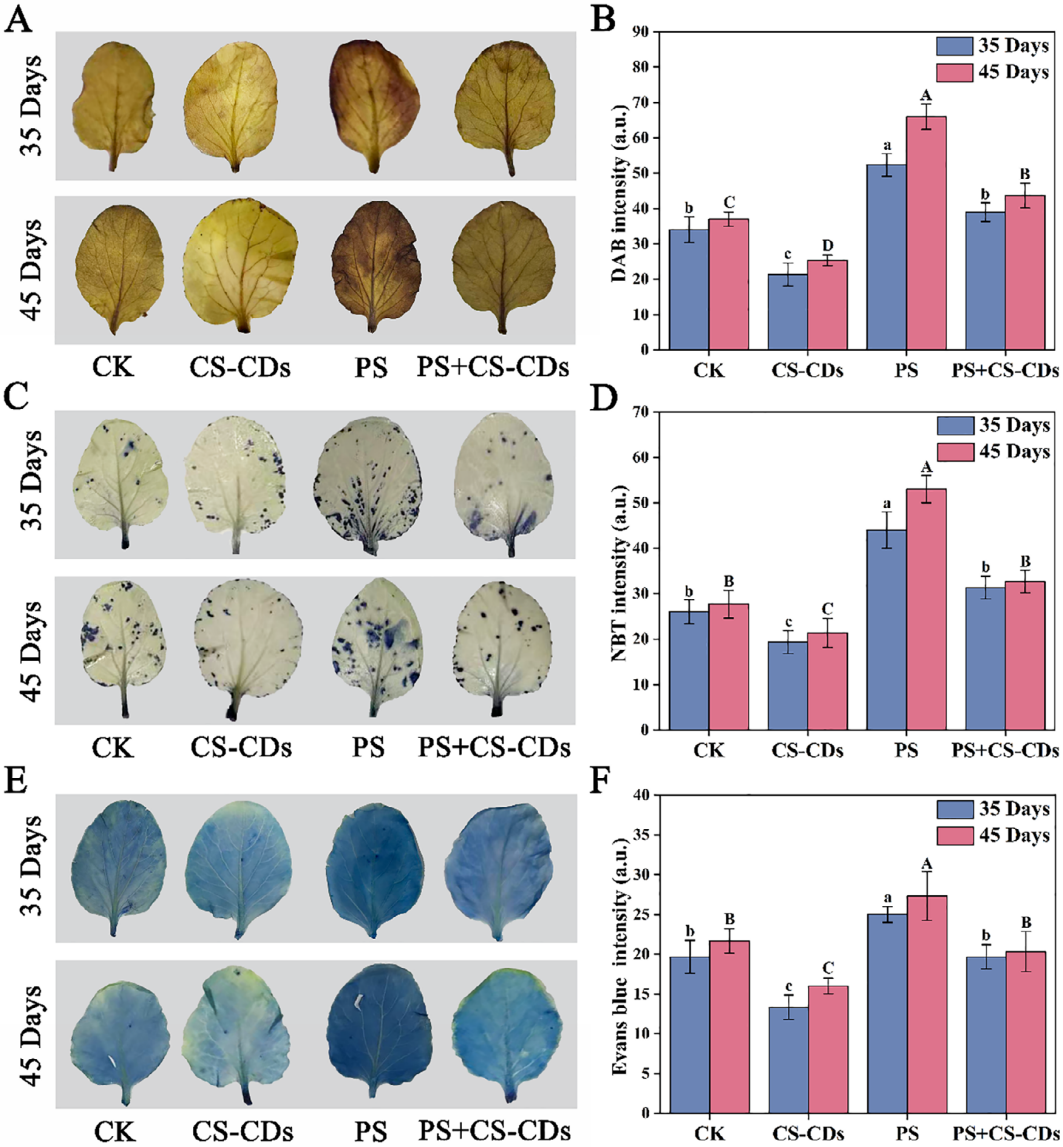
Using CK as the control, the changes in the levels of ROS and the degree of cell damage in the leaves of *Brassica rapa* on the 35th and 45th days were investigated. (A, B) Detection of H_2_O_2_ in *Brassica rapa* leaves by DAB staining. (C, D) Detection of O_2_^•−^ in *Brassica rapa* leaves by NBT staining. (E, F) Detection of cell viability in *Brassica rapa* leaves by Evans blue staining. Data are presented as mean ± standard deviation (n = 6). Bars indicated with different letters indicate significantly different values (*p* < 0.05).

Under stressful conditions, the initial response within plant cells typically manifests as an explosion of ROS [43]. For instance, Li et al. demonstrated that nanoparticles significantly induce the production of ROS within plants, affecting biomass accumulation [4]. To investigate the effects of PS on the oxidative stress and membrane integrity of *Brassica rapa* leaves, we conducted histochemical staining analysis (Figure 5). On the 35th day, the PS group showed significant DAB, NBT, and Evans blue staining, with staining intensities increasing by 53.91%, 69.23%, and 27.10%, respectively, compared to the CK group (*p* < 0.05), indicating that PS induced the accumulation of H_2_O_2_ and O_2_^•−^ in plant leaves and damaged the cell membranes. This might be due to the interaction between PS and plant tissues and related compounds, resulting in oxidative damage and disruption of cell structures [35, 44, 45, 46]. The CS‐CDs treatment significantly reduced the intensities of these three staining methods, with the staining intensities of the PS+CS‐CDs group being 33.83%, 38.36%, and 25.61% lower than those of the PS group by the 45th day (*p* < 0.05), which was consistent with the changes in MDA content (Figure S3A), confirming that CS‐CDs have a sustained role in alleviating oxidative damage.

To cope with oxidative stress, plants activate their antioxidant defense systems to remove excessive ROS, thereby resisting oxidative damage [47]. For example, Wang et al. showed that as the concentration of NPs increased, the contents of ROS and related antioxidant enzymes in *Lettuce* also gradually increased [9]. Compared to the CK group, the activities of peroxidase (POD), superoxide dismutase (SOD), and catalase (CAT) in the PS group gradually increased with the extension of stress time, and compared to the PS group, the activities of the above enzymes in the PS+CS‐CDs group were further increased by 61.78%, 32.34%, and 30.06% (*p* < 0.05, Figure S3C,D), indicating that CS‐CDs may further activate the activities of related antioxidant enzymes. These results collectively confirm that CS‐CDs are effective in eliminating ROS and reducing oxidative damage. CDs have been proven to have significant antioxidant properties, and they can effectively remove ROS and stimulate antioxidant enzyme activities when plants are subjected to stress [48]. This may be attributed to the ability of CDs to act as proton donors to eliminate free radicals and alleviate plant oxidative damage [40]. This characteristic of CDs has also been confirmed in this study (Figure 1E). Therefore, after being transported into the plant, CDs can exert their unique antioxidant capacity, effectively removing ROS and maintaining the redox balance within the cells [25]. Additionally, the surface barrier formed by CS‐CDs provides physical assistance for CDs in alleviating oxidative damage. In conclusion, CS‐CDs effectively remove ROS, regulate the antioxidant enzyme system, and reduce oxidative damage through multiple synergistic mechanisms, thereby maintaining the normal physiological metabolic functions of plants and ultimately promoting the growth of *Brassica rapa*.

### 2.5 CS‐CDs can Effectively Inhibit the Accumulation and Absorption of PS by the Leaves of *Brassica rapa*

**FIGURE 6:**
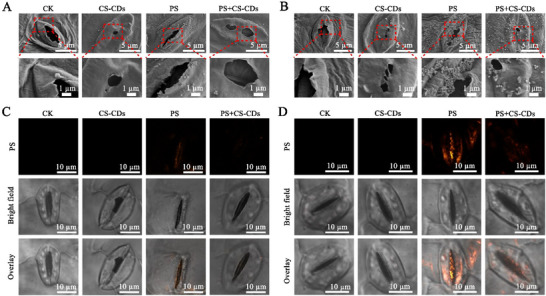
PS accumulation around stomata in *Brassica rapa*. SEM characterization of PS accumulation around stomata in *Brassica rapa* at 35 days (A) and 45 days (B) of growth. Scale bar = 5 µm/1 µm. Laser scanning confocal microscopy characterization of PS accumulation around stomata in *Brassica rapa* at 35 days (C) and 45 days (D) of growth. Scale bar = 10 µm.

The cuticle and stomata of the leaves are the main pathways for M/NPs to enter the plant body [2, 49]. To visually verify the attachment and invasion behavior of PS on the leaf surface, SEM observations showed that obvious PS particle aggregations appeared around the stomata in the PS group on the 35th day, while in the PS+CS‐CDs group, due to the barrier effect of CS‐CDs, the enrichment was significantly reduced (Figure 6A). By the 45th day, the accumulation in the PS group further increased, while the PS+CS‐CDs group still maintained a lower level, confirming that CS‐CDs have a continuous physical barrier function (Figure 6B). Furthermore, in order to investigate whether PS can penetrate into the interior of the stomata, this study conducted fluorescence imaging analysis based on its characteristic of emitting orange fluorescence at a specific wavelength. On the 35th day, weak fluorescence was observed inside and around the stomata in the PS group, indicating that PS had entered the leaf tissue [50], while the fluorescence phenomenon in the PS+CS‐CDs group was almost invisible, confirming that CS‐CDs effectively inhibited the accumulation and transport of PS (Figure 6C). With the extension of time, the fluorescence signal in the PS group further enhanced and extended toward the deeper mesophyll tissue, showing a time accumulation trend (Figure 6D), consistent with the report of Li et al. [2]. While the fluorescence intensity in the PS+CS‐CDs group remained at a low level, further confirming that CS‐CDs have a continuous blocking effect.

**FIGURE 7:**
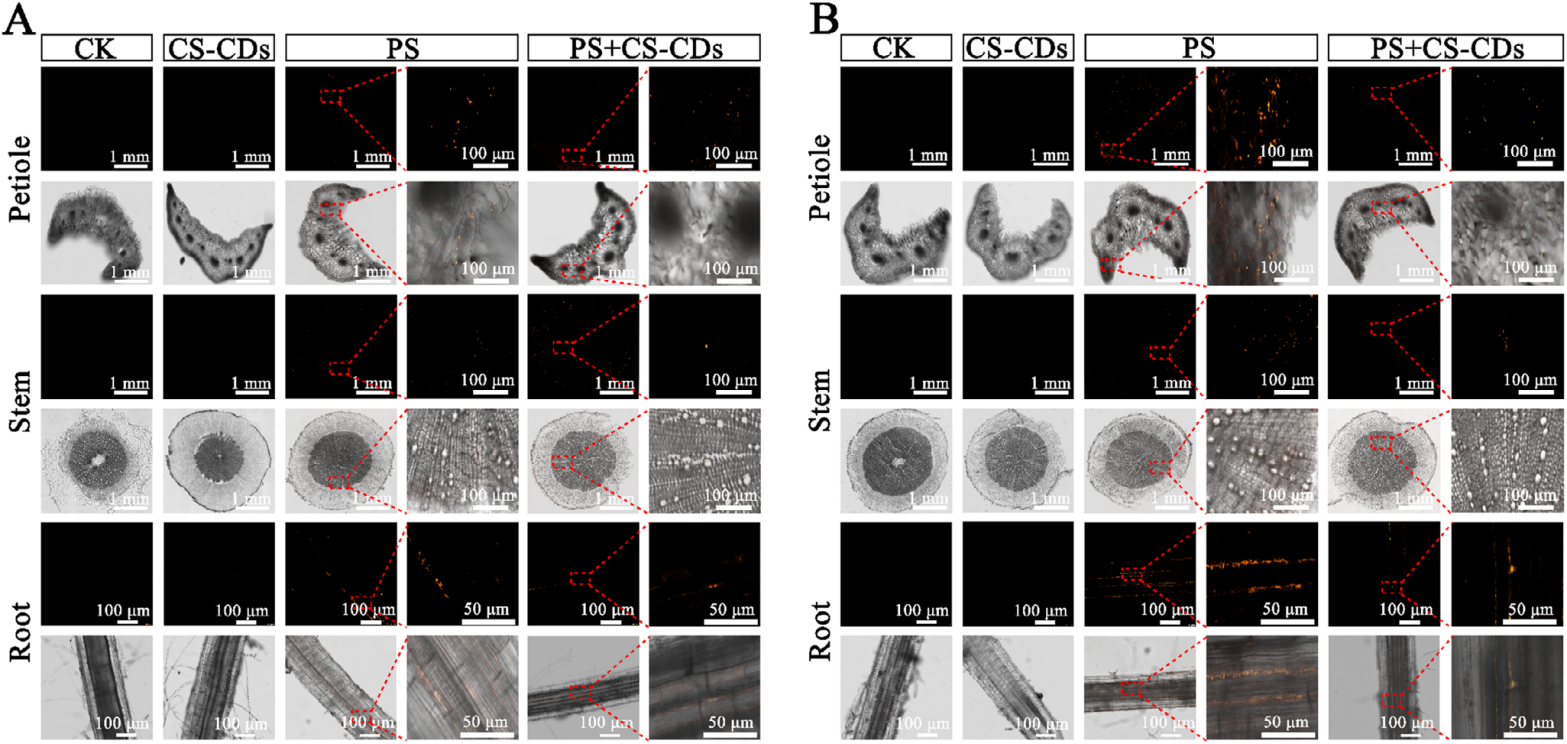
Absorption and transport of PS within plant tissues. PS absorption and transport in the petioles, stems, and roots of *Brassica rapa* at 35 days (A) and 45 days (B) after growth initiation. Scale bar = 1 mm/100 µm/50 µm.

After M/NPs enter the plant body through stomata and cuticle, they will be transported to the roots [51, 52]. Similar results were observed in this study (Figure 7). The observed phenomena in each treatment group were consistent with the distribution results of PS on the leaf surface and inside the stomata. This may be because the physical barrier formed by CS‐CDs on the leaf surface reduced the amount of PS entering the body through the cuticle and stomata, and possibly because of its biological activity, it affected the interaction between the cell wall, cell membrane, and vascular bundles and PS. Moreover, CS‐CDs activated the cuticle synthesis, lignin accumulation, etc. (Figures 9 and 10A), enhanced the mechanical strength of the cell wall, and further hindered the transport process of PS that had already entered the tissue to the vascular bundles.

To further explore whether CS‐CDs also have a mitigating effect on other particulate matter stress in the atmosphere, this study selected widely existing polyethylene NPs (PE) and tire wear particles (TWP) for verification (Figures S4 and S5). The results showed that CS‐CDs treatment significantly reduced the enrichment of these two particulate matter on the leaf surface, indicating that CS‐CDs have a good physical barrier effect for common atmospheric particulate matter, demonstrating its broad‐spectrum application potential for dealing with M/NPs in the atmosphere. However, this does not indicate that CS‐CDs are suitable for dealing with all types of NPs stress. The effectiveness of CS‐CDs for more types of NPs (such as those with different degrees of aging, different densities, and different surface functional groups) still needs to be systematically verified.

### 2.6 CS‐CDs Enrich Beneficial Bacterial Communities in the Leaves to Alleviate the Damage Caused by PS to the Leaves of *Brassica rapa*

**FIGURE 8:**
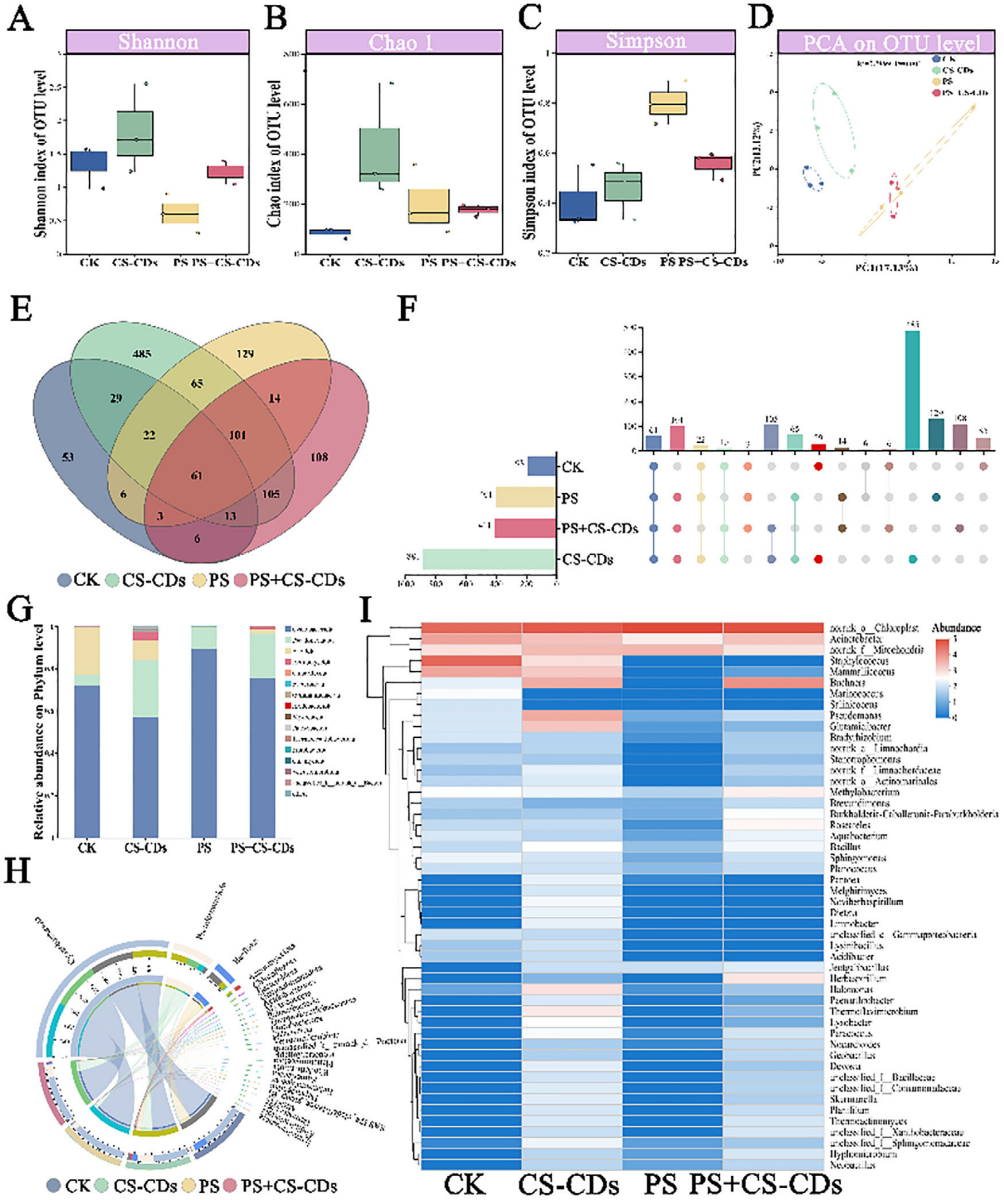
The microbial conditions between the leaves of each group of *Brassica rapa* on the 45th day of culture. (A–C) α‐diversity indices (Shannon, Chao1, and Simpson) comparing the richness and diversity of interfacial bacterial communities among different treatment groups. (D) PCA plot of the overall distribution of bacterial communities at the leaf margin. (E) Venn diagram summarizing the OTU distribution across different samples. (F) Upset plot showing the number of shared and unique OTUs among treatment groups. (G) Relative abundance of bacterial phyla in leaf margin samples under different treatments. (H) Circos plot visualizing the distribution of dominant bacterial taxa at the phylum level. (I) Heatmap showing the relative abundance of dominant bacterial genera across treatment groups, clustered by genus composition.

The surface of the leaf is an important site for the interaction between plants and microorganisms [53]. The inter‐leaf microorganisms play a crucial role in providing nutrients, enhancing disease resistance, and regulating the physiological functions of the leaves [5, 54]. However, atmospheric M/NPs can disrupt the community structure and ecological functions of inter‐leaf microorganisms, thereby posing a threat to the health of plants [55]. To systematically evaluate the effects of CS‐CDs and PS on the inter‐leaf microorganisms of *Brassica rapa*, this study used the Chao 1 index to reflect species richness, and the Shannon and Simpson indices to assess species diversity and evenness [56]. The results showed that the CS‐CDs treatment significantly increased the Shannon index and Chao 1 index, increasing by 26.26% and 395.81%, respectively, compared to the CK group (*p* < 0.05), indicating that it not only increased the number of microbial species but also promoted the balance and stability of the community structure. However, compared to the CK group, the PS treatment increased the Chao 1 index by 137.61%, but significantly decreased the Shannon index by 55.39%, and the Simpson index increased by 98.23% (*p* < 0.05, Figure 8A–C). This indicates that PS stress may lead to abnormal proliferation of a few tolerant groups, although the richness increases, the overall diversity and evenness are significantly reduced, and the community structure becomes more monotonous [57]. This may be attributed to the fact that M/NPs can adsorb environmental toxins or release their own additives, interfering with the genetic activities and interspecies communication of bacteria, thereby posing a threat to the native microbial community [58, 59], and providing new ecological niches for specific microorganisms, promoting their abnormal proliferation [60]. It is worth noting that after combining the application of CS‐CDs on the basis of PS stress (PS+CS‐CDs group), the Shannon index recovered by 103.93% compared to the PS group, and the Simpson index decreased by 30.61%, indicating that CS‐CDs effectively alleviated the loss of microbial diversity caused by PS and restored the ecological balance of the community. PCA analysis further revealed that the microbial community structure was significantly separated among the treatment groups (*p* < 0.05, Figure 8D), indicating that both CS‐CDs and PS specifically regulated the composition of interleaf microorganisms, which might be achieved by inducing differential enrichment of different functional groups [5]. The analysis of Venn plots and Upset plots supports this conclusion (Figure 8E,F): The number of unique OTUs in the CS‐CDs group was the largest (485), which was much higher than that in the PS group (108) and the PS+CS‐CDs group (129) (Figure 8E,F), indicating that CS‐CDs has a significant ability to promote the colonization of specific beneficial bacteria (*p* < 0.05), while PS treatment changed the original community structure and induced the enrichment of specific tolerant populations. It has restricted the ecological niche width of microorganisms [5]. At the microbial community level, the surface of the *Brassica rapa* leaves was dominated by *Cyanobacteriota*, *Pseudomonadota*, *Bacillota*, *Actinomycetota* and *Chloroflexota* (Figure 8G,H). In this study, it was observed that the abundance of *Cyanobacteriota* was the highest in the PS treatment group, with the order being PS > PS+CS‐CDs > CK > CS‐CDs. This trend is consistent with the conclusion of Shi et al. in their study of applying NPs to tomato leaves [5]. *Cyanobacteriota*, as a photosynthetic autotrophic group, have competitive advantages in carbon and nitrogen fixation and stress adaptation [61, 62, 63]. This might be due to the microenvironment changes caused by PS, which inhibit sensitive bacterial communities and create conditions for their proliferation, thereby leading to a decline in community diversity. In contrast, the CS‐CDs treatment alleviated the impact of PS on the microbial community to a certain extent. Among the bacterial phyla, such as *Pseudomonadota*, *Actinomycetota*, and *Chloroflexota*, the richness of the PS+CS‐CDs group was higher than that of the PS group, indicating that CS‐CDs helped maintain the abundance of beneficial microorganisms in the leaf microenvironment. Previous studies have shown that *Pseudomonadota* has the ability to fix nitrogen and can convert nitrogen in the air into ammonia, providing nitrogen sources for plants [64]. *Actinomycetota* can synthesize plant hormones such as auxins, promoting plant growth and development, and can degrade difficult‐to‐degrade organic polymers, promoting nutrient cycling [56]. *Chloroflexota* participates in the degradation of organic compounds such as carbohydrates and lipids on the leaf surface, promoting the carbon cycle process. The abundance of these beneficial bacterial phyla increased in the PS+CS‐CDs and CS‐CDs groups, indicating that CS‐CDs not only did not have a negative impact on the carbon‐nitrogen cycle of plant leaves but also might promote their growth by providing the nutrients needed by surface microorganisms, thereby alleviating the damage caused by PS to the leaf microecology. This change in microbial community structure is consistent with the aforementioned photosynthetic parameters (Figure 4F–I) and ROS and antioxidant enzyme indicators (Figure 5), further confirming the positive role of CS‐CDs in alleviating PS stress, maintaining leaf photosynthetic function and redox balance. Figure 8I shows the results of the top 50 bacterial genera enriched in each treatment group. It indicates that the CS‐CDs group significantly enriched numerous beneficial bacterial genera (*p* < 0.05), while the PS group significantly reduced the enrichment level of these beneficial bacterial genera (*p* < 0.05). The CS‐CDs group significantly enriched several bacterial genera with clear beneficial or stress‐resistant functions (*p* < 0.05), such as *Pseudomonas*, *Pantoea*, and *Devosia*. For instance, multiple strains of *Pseudomonas* have antagonistic effects against *Fusarium* [65], and some strains can synthesize iron carriers and ROS, participating in the regulation of the leaf surface microenvironment [66]; *Pantoea* can produce plant hormones to promote plant growth and inhibit tomato gray mold [67]. Notably, in the PS+CS‐CDs group, due to the possibility that CS‐CDs may reduce the adsorption of PS by the leaf surface, the abundance of several beneficial bacterial genera was restored, such as *Methylobacterium*, *Bacillus*, and *Sphingomonas*. Among them, *Methylobacterium* can utilize the methanol released by the leaves as a carbon source to promote plant growth; *Bacillus* and *Sphingomonas* possess the ability to resist abiotic stresses such as drought and osmotic stress, and can repair cell damage caused by ROS [68]. These results indicate that CS‐CDs help to construct a beneficial bacterial community with stress‐resistant and promoting functions at the leaf interface. To sum up, although PS stress may induce the proliferation of specific tolerant flora, it generally leads to a decrease in interfoliar microbial diversity and an imbalance in community structure. CS‐CDs can not only directly promote the enrichment and diversity improvement of beneficial microorganisms, but also effectively repair the damaged flora structure and restore its ecological function in the presence of PS. This study provides a theoretical basis for regulating the interleaf microecology with environmentally friendly materials and alleviating the negative impact of NPs pollution on crops.

### 2.7 The Effects of Different Treatment Groups on the Metabolism of *Brassica rapa* Leaves

**FIGURE 9:**
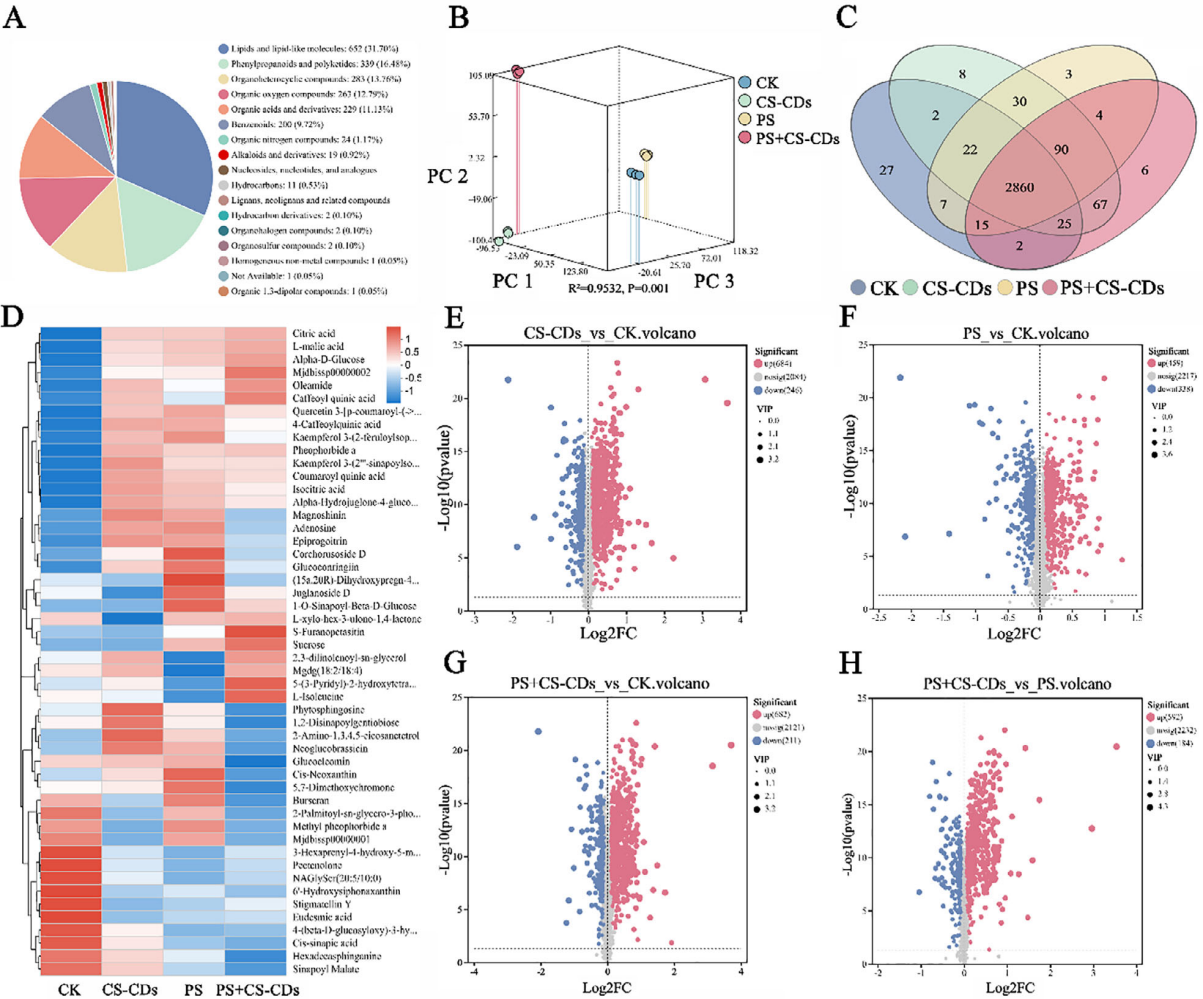
Metabolome analysis of *Brassica rapa* grown to 45 days old. (A) Classification of leaf metabolites. (B) PCA analysis of leaf metabolism. (C) Analysis of Venn diagrams of leaf metabolism. (D) Heatmap of the top 50 leaf metabolism. (E‐H) The volcano map of differential metabolites up and down regulate, where red dots indicate up‐regulated metabolites and blue dots denote down‐regulated metabolites based on the log2FC and log10 (p value). n = 6.

**FIGURE 10:**
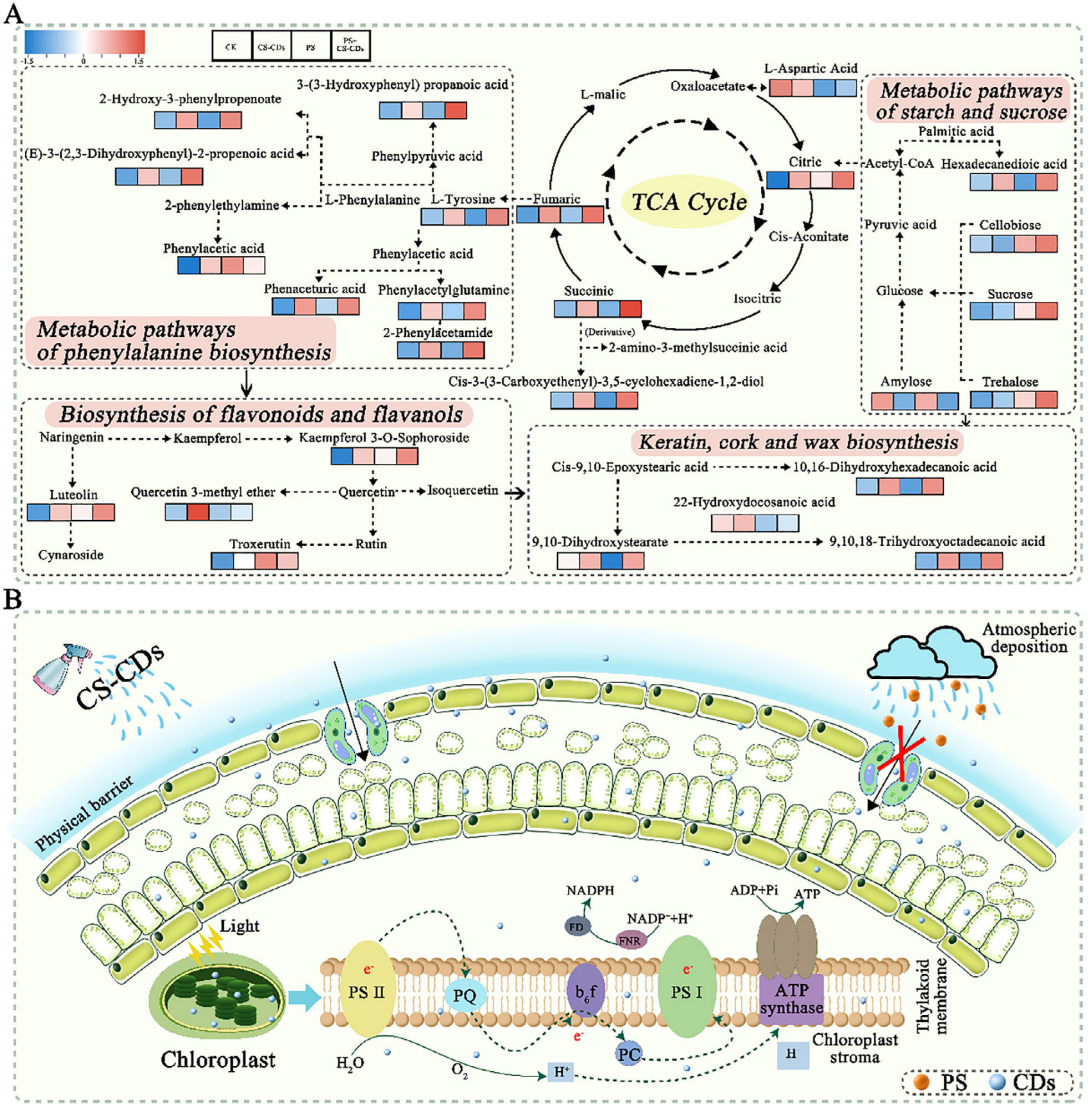
All different treatment groups had significant effects on the metabolic pathways of *Brassica rapa*. (A) The expression levels of representative metabolites in the key metabolic pathways of the four groups of CK, CS‐CDs, PS, and PS+CS‐CDs. n = 6. (B) CS‐CDs form a protective film on the leaf surface, reducing the accumulation and invasion of PS. At the same time, the CDs loaded on them can be absorbed and transported by the leaves, stimulating the plant's own resistance, forming a coordinated protective mechanism from the leaf surface to the inside of the plant, to resist the stress caused by PS.

Plant metabolism is a fundamental process for maintaining life activities, responsible for converting exogenous substances into organic molecules, excreting metabolic wastes, and providing energy support for growth and development [69, 70]. Metabolites, as the end products of enzyme‐catalyzed reactions, can reflect the physiological responses and biochemical regulatory mechanisms of plants under stress [71]. This study conducted metabolomics analysis based on the HMDB database. A total of 2,057 metabolites were identified in each treatment group, belonging to 17 categories. Among them, *Lipids and lipid‐like molecules*, *Phenylpropanoids and polyketides*, *Organoheterocyclic compounds compounds*, *Organic oxygen compounds*, and *Organic acids and derivatives*, etc., account for a relatively high proportion (Figure 9A). *Lipids and lipid‐like molecules* account for the largest proportion among all metabolites. As an important component of the cell membrane structure, their content changes directly affect the stability and function of the cell membrane, and thereby regulate the absorption and transport of nutrients [72]. This result might be due to the fact that PS inhibits lipid accumulation by interfering with the membrane lipid synthesis pathway, while CS‐CDs significantly increase the expression level of such metabolites by reducing the adsorption of PS (*p* < 0.05). Principal component analysis showed that the metabolic profiles of each treatment group were significantly separated (*p* < 0.05), indicating that CS‐CDs, PS, and PS+CS‐CDs all had significant effects on the metabolic composition of plants (*p* < 0.05, Figure 9B). Venn diagram analysis further revealed that CS‐CDs treatment increased the number of metabolites to 3,104, compared with 3,031 in the PS group, 3,069 in the PS+CS‐CDs group (Figure 9C). It indicates that CS‐CDs can enhance metabolic diversity, while PS shows an inhibitory trend, which is consistent with the negative effects of M/NPs on the metabolism of *Zea mays* [69] and *Lactuca sativa* [73]. Analysis of the top 50 enriched metabolites indicated that PS altered the distribution pattern of metabolites in *Brassica rapa* (Figure 9D). For example, *Methyl pheophorbide a* is a key intermediate product in the chlorophyll degradation pathway and has photosensitive properties. Its excessive accumulation can promote apoptosis [74, 75]. This study shows that this metabolite has the highest relative content in the PS group, and its expression levels are in the order of PS > CK > PS+CS‐CDs > CS‐CDs. This might also be one of the direct reasons for the yellowing of the leaves and the decrease in chlorophyll content of the PS group's *Brassica rapa*. Furthermore, the expression of *1‐O‐Sinapoyl‐Beta‐D‐Glucose* involved in the synthesis of phenolic acids and flavonoids was enhanced in the PS group (PS+CS‐CDs > PS > CK > CS‐CDs), which might be attributed to the activation of the plant defense system by oxidative stress [76]. And CS‐CDs, due to their own antioxidant capacity, reduce the activation demand of plants for this pathway. Through volcanic map analysis, a total of 3,014 metabolites were identified (Figure 9E–H). Compared with CK, CS‐CDs, PS, and PS+CS‐CDs were up‐regulated by 684, 459, and 682 of metabolites, respectively, and down‐regulated by 246, 338, and 211 metabolites, respectively. It is worth noting that compared with the PS group, the PS+CS‐CDs group up‐regulated 592 metabolites and down‐regulated 184 metabolites. This indicates that PS can lead to the down‐regulation of metabolite expression levels on the leaf surface and may disrupt metabolic homeostasis, while CS‐CDs can effectively reverse this trend. KEGG pathway enrichment analysis revealed that different treatments activated different metabolic pathways, reflecting significant differences in their regulatory patterns (*p* < 0.05, Figure S6). In the CS‐CDs and PS+CS‐CDs groups, the biosynthetic pathways of *keratin, cork, and wax biosynthesis* were significantly enriched (*p* < 0.05). The key metabolites in this pathway, such as *10,16‐Dihydroxyhexadecanoic acid*、*9,10‐Dihydroxystearate*、*22‐Hydroxydocosanoic acid*、*9,10,18‐Trihydroxyoctadecanoic acid*, etc., were significantly increased in expression in the CS‐CDs and PS+CS‐CDs groups (*p* < 0.05), but significantly decreased in the PS group (*p* < 0.05, Figure 10A). Previous studies have pointed out that the products of this pathway are an important component of the epidermal stratum corneum [77], which can enhance the physical barrier function of leaves and reduce the adhesion and invasion of M/NPs [9]. However, no enrichment of this pathway was observed in the PS group, which might be related to the destruction of the waxy structure by PS [78]. Furthermore, CS‐CDs also activate the *biosynthesis of phenylalanine*, *tyrosine, and tryptophan*, as well as the *phenylalanine metabolism* pathway (Figure S6A). The expression levels of key metabolites such as *L‐Tyrosine*, *3‐(3‐Hydroxyphenyl) propanoic acid*, *2‐Hydroxy‐3‐phenylpropenoate*, *Phenylacetylglutamine*, *2‐Phenylacetamide*, *LuteolinKaempferol 3‐O‐Sophoroside* were upregulated (Figure 10A). These metabolites can effectively promote the synthesis of secondary metabolites such as lignin and flavonoids [79, 80]. Studies have shown that lignin can enhance the hardness of cell walls [79], while flavonoids can regulate auxin synthesis [81] and participate in stress response and antioxidant processes [82, 83], thereby helping to improve the stress resistance and nutritional value of plants. In contrast, PS treatment significantly enriched pathways related to stress responses (*p* < 0.05), such as *glutathione metabolism* and *linoleic acid metabolism*. The activation of *glutathione metabolism* is a typical response of plants to oxidative stress [84], which is consistent with the changing trend of antioxidant enzyme activity in Figure S3B–D. *Linoleic acid metabolism* is closely related to membrane lipid peroxidation [85, 86], suggesting that PS may have caused damage to the membrane system. Although *glutathione metabolism* was still enriched to a certain extent in the PS+CS‐CDs group, the metabolic focus shifted significantly toward the synthesis of defense substances, manifested as the synergistic accumulation of keratin waxy metabolites and flavonoids. This might be a manifestation of the plant's self‐regulation by moderately activating the antioxidant system. In the direct comparative analysis of the PS+CS‐CDs group and the PS group, pathways such as *keratin, cork, and wax biosynthesis*, *D‐amino acid metabolism*, and *phenylpropane metabolism* showed significant enrichment (*p* < 0.05). These metabolic processes are closely related to the stress resistance of plants and can synergistically enhance the physiological adaptability of plants to stressful environments [9, 82, 83]. This indicates that CS‐CDs can stably regulate the above metabolic network and effectively promote the accumulation of defense‐related substances and functional components in *Brassica rapa*. In addition, each treatment significantly affected the metabolism of starch and sucrose as well as the changes in metabolites related to the TCA cycle (*p* < 0.05, Figure 10A). PS+CS‐CDs treatment significantly increased the sucrose content in *Brassica rapa* (*p* < 0.05). Sucrose can not only serve as a precursor of secondary metabolites but also provide a carbon skeleton for other organic molecules [87]. Its accumulation helps enhance the plant's ability to alleviate PS stress. Organic acids in the *TCA cycle* (including *fumaric acid*, *succinic acid*, and *citric acid*) play a significant role in regulating cellular redox balance, pH, and ionic homeostasis, jointly supporting the normal growth and development of plants [87]. This study found that the expression levels of these organic acids in both the CS‐CDs and PS+CS‐CDs groups were higher than those in the PS group, further indicating that CS‐CDs can effectively enhance the physiological relief ability of *Brassica rapa* to PS stress.

As a nanomaterial protection strategy, CS‐CDs can form a stable and dense protective film on the leaf surface to improve the smoothness of the leaf, thereby significantly reducing the accumulation of PS in *Brassica rapa* leaves (*p* < 0.05). In addition, the CDs loaded on CS‐CDs will be transported to the roots after entering the plant through the leaves (Figure 3). With its excellent antioxidant capacity and light conversion characteristics, it effectively reduces oxidative damage and improves photosynthetic efficiency, thereby promoting the accumulation of beneficial metabolites such as *10,16‐dihydroxyhexadecanoic acid* and activating the phenylpropanoid metabolic pathway, promoting the accumulation of secondary metabolites such as flavonoids and lignins, and strengthening the cell wall structure. Moreover, CDs may also serve as a carbon source for inter‐leaf microorganisms, promoting the increase in the diversity of inter‐leaf microorganisms (Figure 10B). This enables the function of CDs to expand from simple antioxidant effects to forming a more dense physical barrier with CS, thus more effectively responding to the dual pressures of NPs on the leaf surface and inside the plant. This dual protection mechanism compensates for the functional limitations of a single material. However, the current research results do not fully prove that CS‐CDs can withstand the stress of NPs in the atmosphere of real environments. In the future, we will further explore the wide applicability of CS‐CDs and its related mechanisms, and at the same time, conduct field experiments to further test their persistence and application strategies in actual environments.

## 3 Conclusion

This study systematically investigated the effect and mechanism of CS‐CDs in alleviating the stress caused by PS in the atmosphere on *Brassica rapa*. The results showed that CS‐CDs could form a uniform and dense persistent protective film on the surface of *Brassica rapa* leaves, significantly reducing the attachment of PS on the leaf surface and its invasion through the stomata pathway, without causing any damage to the leaf structure. At the same time, the CDs loaded on CS‐CDs could be effectively absorbed by the leaves and transported long distances to organs such as stems and roots. While exerting antioxidant enzyme activity to remove the excessive ROS induced by PS, they cooperated with CS to activate defense pathways such as *cutin, wax, betulinic acid biosynthesis*, and *phenylpropanoid metabolism*, thereby enhancing the intrinsic stress resistance of plants at the system level. Based on these mechanisms, CS‐CDs effectively alleviated the growth inhibition (compared with the PS group, the fresh weight of the PS+CS‐CDs group increased by 26.17%, and the dry weight increased by 30.77%) and photosynthetic damage (compared with the PS group, the Pn of the PS+CS‐CDs group increased by 49.21%) caused by PS stress, significantly reducing the oxidative stress levels (ROS and MDA content decreased). Moreover, CS‐CDs also optimized the structure of the leaf‐associated microbial community, enriched beneficial bacterial groups such as *Pseudomonadota* and *Actinomycetota*, and restored the imbalance of the leaf‐associated microbial community caused by PS, thereby enhancing the health level of plants at the microbial level. This study systematically revealed the integrated mechanism of CS‐CDs from physical barrier at the leaf surface, physiological regulation, microbial community optimization, and metabolic pathway activation, achieving an expansion from a single pollutant barrier to multi‐target collaborative improvement (pollutant barrier, biomass increase, microbial restoration, intrinsic defense activation). This study provides a new strategy for reducing the accumulation of atmospheric M/NPs in crops and their transfer along the food chain, and also provides a new theoretical basis for the application of multifunctional nanomaterials in sustainable agriculture. Future research will further verify the alleviating effect of CS‐CDs on other types of microplastics in a natural environment, and conduct systematic studies in complex polluted environments to assess its application value in the natural environment.

## 4 Experimental Section

### 4.1 Material

CS (deacetylation ≥ 95%, viscosity of 100–200 mpa.s and average molec regular weight of 150 kDa), glacial acetic acid (purity ≥ 99.5%, MW = 60.05), DPPH, both purchased from Macklin Biochemical Co., Ltd. (Shanghai, China). The PS fluorescent microspheres used in the experiment were procured from Tianjin Saierqun Technology Co., Ltd. (Tianjin, China). Regarding the characterization of PS, please refer to Figure S7. The excitation peak wavelength (λ_ex) and emission peak wavelength (λ_em) of these microspheres were 540 and 580 nm, respectively. The *Brassica rapa* seeds, PE, and TWP were purchased from the local market (Hefei, China). The methods and results for characterizing PE and TWP are presented in Text S5 and Figures S8 and S9. The *Cercis chinensis* pods were taken from the campus of Anhui Agricultural University (Hefei, China). All other chemicals and reagents employed were of analytical grade.

### 4.2 Synthesis and Characterization of CDs

#### 4.2.1 Synthesis of CDs

First, the *Cercis chinensis* pods were dried in an oven at 50°C, then ground into powder to serve as the carbon source for synthesizing CDs. The synthesis of CDs followed the method reported by Liu et al. [12]. Briefly, 1 g of *Cercis chinensis* pods powder was dissolved in 60 mL of deionized water and stirred magnetically for 20 min to ensure complete dissolution and thorough mixing. Subsequently, the mixture was transferred to a high‐pressure reactor and reacted at 190°C for 6 h. After reaction completion, the mixture was allowed to cool naturally to room temperature (approximately 25°C), yielding a yellowish‐brown solution. This solution was centrifuged at 8000 rpm for 15 min, and the supernatant was filtered through a Whatman nylon film filter (pore size 0.22 µm, diameter 25 mm) to obtain the CDs solution. Finally, the resulting brownish‐yellow solution was freeze‐dried and stored at 4°C for subsequent analysis and use.

#### 4.2.2 The characterization of CDs and PS

Detailed information on CDs and PS characterization is in Text S1 of the Supporting Information.

### 4.3 The Antioxidant Capacity of CDs and CS‐CDs

Following the methodology of Liu et al. [12], the antioxidant capacity of CDs and CS‐CDs solutions at various concentrations (12.5, 25, 50, 75, and 100 µg/mL) was evaluated using the DPPH method. The procedure was as follows: 10 mL of freshly prepared DPPH working solution was taken, to which pre‐determined concentrations of CDs and CS‐CDs solutions were added. The mixtures were thoroughly mixed and allowed to stand at room temperature, protected from light, for 30 min. Subsequently, the absorbance values of each reaction system were measured at 517 nm using a UV–vis spectrophotometer to evaluate their radical scavenging capacity.

### 4.4 Design of the Cultivation and Experimental Process of *Brassica rapa*

#### 4.4.1 Cultivation of *Brassica rapa*

Building upon traditional cultivation methods, this study systematically optimised the planting process for *Brassica rapa*. First, seeds underwent surface disinfection with a 0.02% sodium hypochlorite solution for 20 min, followed by repeated rinsing with distilled water. These were then sown in seedling trays for germination. After two days of germination, uniformly sprouted seedlings were selected, with two plants per pot transplanted into containers holding approximately 200 g of mixed substrate. This substrate mixture comprised coconut husk fibre, standard garden soil, and nutrient soil in a 7:2:1 volume ratio, with pots measuring 10 cm in diameter. Following transplanting, plants were cultivated in a light‐controlled chamber under conditions of 26°C temperature, 60% relative humidity, and a photoperiod of 16 h light/8 h darkness.

#### 4.4.2 Design of Pre‐Experiment Process for PS Concentration

For detailed information on the concentration setting of the PS solution in the pre‐experiment and the method of adding the PS solution to the plant leaf surface, please refer to Text S2. According to the pre‐experiment results shown in Figure S10, when the PS solution concentration was 150 µg·d^−^^1^ (per plant), the enrichment degree of PS in the stomata and the surrounding area of the *Brassica rapa* leaves was the strongest.

#### 4.4.3 Design of Preliminary Experiments to Determine the Appropriate Concentrations of CS‐CDs

For the detailed information on the pre‐experiment plan for the concentration of CS‐CDs solution and the method of adding the CS‐CDs solution, please refer to Text S3. According to the preliminary experimental results shown in Figure S11, when 0.2 g of CS and 0.2 g of CDs are dissolved in 25 µL of acetic acid in 100 mL of deionized water, compared with other concentrations, this concentration of CS‐CDs solution can uniformly adhere to the plant leaves, and while ensuring that it can effectively reduce the accumulation of PS in the plant leaves, it has the least impact on the respiration of leaf stomata.

#### 4.4.4 Experimental Process Design

This experiment began on the 25th day after the *Brassica rapa* seeds were transplanted into the planting pots and was started to be conducted. Six *Brassica rapa* seedlings of the same growth condition were selected and set up into four treatment groups. Each group contained six *Brassica rapa* seedlings. The highest concentration of M/NPs in the dust of the urban atmosphere in China was 350 mg·g^−^^1^ [9], but the content of M/NPs in the atmosphere fluctuates. Studies have shown that the retention amount of microplastics by plant leaves is positively correlated with the concentration of microplastics in the atmosphere and the growth time of the plant [2]. Based on the calculation formula for “leaf microplastic retention” established by Wang et al. [9]: “Leaf microplastic retention = Plant dust retention ability × Leaf area × Microplastic concentration in dust”, the maximum retention amount of microplastics by a single plant leaf can be estimated to be approximately 116 µg·day^−^^1^. Taking the average leaf area of the *Brassica rapa* plants at the 25th day of growth as the benchmark, the corresponding daily deposition flux of PS was 23.2 mg·m^−^^2^·day^−^^1^. To ensure that the plants exhibit significant and measurable physiological responses under controlled and repeatable experimental conditions, thereby systematically analyzing the alleviation mechanism of CS‐CDs and evaluating their maximum protective capacity under extreme pollution conditions, this study combined the results of the pre‐experiment and set the PS exposure dose for each *Brassica rapa* plant at 150 µg·day^−^^1^, corresponding to a daily deposition flux of 30 mg·m^−^^2^·day^−^^1^, as the extreme deposition model for the subsequent experiments.

Given the significant role of precipitation (including rain and snow) in the atmospheric deposition process, based on previous research methods [2, 4, 5, 9, 11], this study adopts the wet deposition method to construct a controllable, efficient and repeatable extreme experimental model, in order to minimize experimental interference and deeply explore the adhesion, penetration of NPs on *Brassica rapa* leaves and the stress response mechanism of the plants. At the same time, it references the method of Wang et al. to simulate the continuous stress of NPs on the leaves of *Brassica rapa* in the atmosphere [9]. The specific operation steps are as follows: Dissolve 150 µg of PS in 100 µL of distilled water and ultrasonicate for 30 min. This amount is the daily usage for each plant. To simulate a continuous stress state, use a pipette to evenly drip 50 µL of the PS solution (with a concentration of 1.50 µg/µL) onto the proximal surface of the third leaf of the *Brassica rapa* every 12 h. This group is named the PS group. This method ensures that the dose of NPs stress on each plant is precisely controllable while simulating the continuous accumulation process of pollutants on the leaf surface [11], which is conducive to deeply exploring the stress response of *Brassica rapa* to NPs stress under repeatable experimental conditions. The CK group is applied with 50 µL of ultrapure water on the leaves at the same time point. In the CS‐CDs group, combined with the pre‐experiment results in Figure S3, 10 mL of CS‐CDs (0.2% w/v each) solution is uniformly sprayed onto each plant's leaves every five days, and the CK group and the PS group are sprayed with the same amount of distilled water.

In addition, to investigate the alleviation effect of CS‐CDs on PS stress, a PS+CS‐CDs group is set up: this group drips PS solution (the same as the PS group) every 12 h and sprays 10 mL of CS‐CDs solution (the same as the CS‐CDs group) every five days. During the application of the solution, all treatment groups cover the soil with aluminum foil to prevent the solution from penetrating the roots and interfering with the experimental results. Different treatment groups' leaves from the same part of the plants are collected for subsequent research on the 35th and 45th days of *Brassica rapa* growth.

### 4.5 Plant Growth Physiological Index Data Measurement

The detailed testing methods for plant‐related physiological indicators are provided in Text S4.

### 4.6 PE and TWP Are Enriched and Accumulated in Plant Leaves

Regarding the observation of the enrichment of PE and TWP on the surface of the *Brassica rapa* leaves, the specific experimental design is described in Text S5.

### 4.7 Interleaf Microbiological Detection

On day 45 of cultivation, leaf samples of *Brassica rapa* were collected from each treatment group. Total DNA from the leaf‐epidermal microbiota was extracted from the surface of fresh leaves using the TruSeq DNA Sample Preparation Kit. The resulting DNA was stored at −80°C for subsequent use. For PCR amplification targeting the V3‐V4 variable region of the bacterial 16S rRNA gene, primers 338F (ACTCCTACGGGAGGCAGCAG) and 806R (GGACTACHVGGGTWTCTAAT) were employed. The amplified products underwent high‐throughput sequencing on the Illumina MiSeq platform (USA). Raw data were processed on the Majorbio cloud platform and analysed against the latest database version (silva138.2/16s_bacteria).

### 4.8 Metabolomics Detection of Leaves

Referring to the method of Li et al. [56], the leaf samples of *Brassica rapa* from different treatment groups growing up to the 45th day were collected for LC‐MS/MS metabolomics analysis. Sample preparation and bioinformatics analysis were carried out by Shanghai Majorbio Co., Ltd. (http://wwwmajorbiocom/) in accordance with standard procedures. To obtain a partial list of LC‐MS/MS raw data, through the human metabolome database (HMDB, http://www.hmdb.ca/) and compare the Metlin database (https://metlin.scripps.edu/), to identify metabolites. The matched metabolite data were subsequently processed and analyzed.

### 4.9 Statistical Analyses

The experimental data were all derived from at least three independent replicates, and the results were expressed as mean ± standard deviation. The significance of the differences between groups was determined by one‐way analysis of variance (ANOVA) and Duncan's multiple comparison test using SPSS 25.0 software, and the significance level was set at *p* < 0.05. All charts were drawn using Origin 2024 software.

## Author Contributions

Beibei Zhao: Writing – original draft, Software, Methodology, Investigation, Formal analysis, Conceptualization. Mei Li: Data curation. Mengjiao Fan, Chuanhuan Liu, Jie Jiang, and Jia Song: Investigation and Software. Yingzhu Liu: Writing – review and editing, Validation, Supervision, Resources, Project administration, Methodology, Funding acquisition, Conceptualization.

## Conflicts of Interest

The authors declare no conflicts of interest.

## Supporting information




**Supporting File**: advs75278‐sup‐0001‐SuppMat.docx.

## Data Availability

The data that support the findings of this study are available on request from the corresponding author. The data are not publicly available due to privacy or ethical restrictions.
