# Supplementary material for: Chitosan‐Carbon Dot Composite Materials Form a Leaf Surface Barrier to Mitigate the Enrichment and Invasion of Nanoplastics: From Leaf Interface to Systemic Response
Source: Adv Sci (Weinh). 2026 Apr 16;13(39):e75278. doi: 10.1002/advs.75278 (PMC13334960; doi:10.1002/advs.75278)

**Supporting information**

**Chitosan-Carbon Dot Composite Materials Form a Leaf Surface Barrier to Mitigate the Enrichment and Invasion of Nanoplastics: From leaf Interface to Systemic Response**

**Beibei Zhao^a^, Mei Li^a^, Mengjiao Fan^a^, Chuanhuan Liu^a^, Jie Jiang^a^****, Jia Song^a^, Yingzhu Liu^a*^**

^a^School of Forestry and Landscape Architecture, Anhui Agricultural University, Hefei 230036, China

*Corresponding author: Tel: +86 18810980506; yingzhu_liu@ahau.edu.cn (Yingzhu Liu)

**Text S1. The characterization of CDs and PS.**

Transmission electron microscope (TEM) (HT7700, Hitachi Production Co., Ltd., Japan) was used to observe the micromorphology of CDs and determine its size and morphological characteristics. The particle size distribution and average particle size were analyzed statistically by Image J software. In terms of optical properties, fluorescence spectrophotometer (F-2710, Hitachi, Japan) and UV-VIS spectrophotometer were used to measure the fluorescence response and light absorption characteristics, respectively. The elemental composition and functional group structures of CS, CDs, and CS-CDs were analysed using a Fourier transform infrared spectrometer (Nicolet iS50, Massachusetts, USA) within the wavenumber range of 4000–500 cm⁻¹. The chemical state of the elements was analyzed by K-alpha X-ray photoelectron spectrometer (XPS) (Thermo Scientific, USA). SEM (S-4800, Hitachi Production Co., Ltd., Japan) was used to observe the micromorphology of PS and determine its size and morphological characteristics.

**Text 2. Design of Pre-experiment Process for PS Concentration.**

Regarding the addition method and concentration of PS, we referred to the experimental methods of Wang et al. and adopted the wet deposition method to simulate the harm that PS brings to plants through atmospheric deposition [1]. Specifically, PS was dissolved in deionized water, and then it was added to the proximal surface of the *Brassica rapa* leaves using a dropper. We selected *Brassica rapa* plants that had grown uniformly for the 30th day and divided them into 5 groups, with 6 *Brassica rapa* seedlings in each group. The concentration gradient of PS was set at 0 μg·d⁻¹ (CK group), 50 μg·d⁻¹, 100 μg·d⁻¹, 150 μg·d⁻¹, and 200 μg·d⁻¹. On the 31st day, different concentrations of PS were dissolved in 100 μL of deionized water and then ultrasonicated for 20 minutes before being dripped onto the proximal surface of the *Brassica rapa* leaves. This was done once every 12 hours, with 50 μL added each time. The CK group was dripped with an equal amount of deionized water, and the process lasted for 15 days. During this period, the growth of *Brassica rapa* plants in different treatment groups was closely observed. When the *Brassica rapa* plants grew to the 45th day, the enrichment of PS around the stomata of different treatment groups of *Brassica rapa* was observed using scanning electron microscopy and laser electron microscopy. The results show that when the concentration of PS on each plant leaf is 150 μg·d⁻¹, the enrichment of PS in the leaf stomata and surrounding tissues is the most significant.

**Text S3.** **Design of preliminary experiments to determine the appropriate concentrations of CS-CDs.**

Regarding the determination of the concentrations of CS-CDs, the concentration of CS was based on the experimental concentration reported by Khodadadi et al. [2], while the concentration of CDs was set according to the research of Liu et al. [3]. To ensure that CS and CDs exert a synergistic effect in the experiment, each group was prepared at a 1:1 ratio. *Brassica rapa* seedlings that had grown to the 30th day and were uniform in growth were selected and divided into five groups, with 6 plants in each group. The CS-CDs concentration gradients were as follows: 0% CS + 0% CDs (CK group), 0.1% CS + 0.1% CDs, 0.2% CS + 0.2% CDs, 0.5% CS + 0.5% CDs, 1.0% CS + 1.0% CDs. Starting from the 35th day, the solutions of CS-CDs at different concentrations were evenly sprayed onto the leaf surfaces of the plants every five days, with 10 mL sprayed per treatment each time to form a protective film on the leaf surface. This treatment was carried out for 15 days, during which the physiological conditions of each group of plants were observed regularly. On the 45th day, SEM was used to observe the film formation of CS-CDs on the leaf surface. The results showed that the treatment group with a concentration of 0.2% CS + 0.2% CDs had the best protective film formation on the leaves and the least adverse effects on plant growth.

**Text S4 Measurement of plant-related physiological indicators**

**Text S4.1 Measurement of plant growth physiological indicators**

Samples were collected from *Brassica rapa* plants on days 35 and 45 of growth. Growth parameters were measured according to the method of Liu et al. [3], and photographs were taken. Specifically, plants were washed and their fresh weight determined immediately using an analytical balance. They were then dried in a 70°C oven to constant weight, after which dry weight was measured. Additionally, plant height, root length and root tip count were quantified using ImageJ software and its SmartRoot plugin.

**Text S4.2 The adhesion of CS-CDs on the leaf surface of *Brassica rapa***

**Text S4.2.1. Observation of the cross-section of the leaf**

To characterise the adhesion of CS-CDs on *Brassica rapa* leaf surfaces, SEM samples were prepared following the method of Wang et al. [1]. Briefly, leaf samples were freeze-dried, subjected to 60 seconds of gold sputtering to enhance conductivity, sectioned perpendicular to the midrib to obtain leaf cross-sections, and analysed via SEM imaging at 20 kV acceleration voltage under high vacuum conditions.

**Text S4.2.2. The distribution of CS-CDs on the blade surface**

The distribution uniformity of CS-CDs on the leaf surface was analysed using an energy dispersive X-ray spectroscopy (EDS, Ametek, US). Following freeze-drying of the PS+CS-CDs group leaves, EDS scanning was performed alongside reference membranes fabricated from pure CS-CDs. Uniformity was assessed by comparing distribution spectra of identical elements.

**Text S4.3 Determination of photosynthetic parameters**

On days 35 and 45 of *Brassica rapa* growth, key photosynthetic parameters of leaf tissue across treatment groups were measured using the CIRAS-3 portable photosynthesis measurement system, following the methodology of Li et al. [4]. These parameters included net photosynthetic rate (Pn), transpiration rate (Tr), stomatal conductance (Gs), and intercellular CO₂ concentration (Ci).

**Text S4.4 The distribution of PS on the leaf surface of *Brassica rapa***

**Text S4.4.1 SEM characterizes the distribution of PS**

Following the methodology of Wang et al. [1], cabbage leaves grown under different treatments for 35 and 45 days were freeze-dried. Subsequently, SEM observations were conducted under high vacuum conditions to examine the aggregation state of PS within leaf stomata and their surrounding regions.

**Text S4.4.2 The distribution of PS was characterized by laser confocal microscopy**

Following the methodology of Wang et al.[1], fresh leaf epidermal samples from different treatment groups grown to 35 and 45 days were placed on microscope slides. Ultra-pure water was added dropwise, followed by sealing with a coverslip. Observations were subsequently conducted using a LSCM (STELLARIS5, Leica, Germany), with excitation set at 540 nm and emission at 580 nm.

**Text S4.5 The absorption and transport of PS and CDs in plants**

**Text S4.5.1 The absorption and transport of PS in *Brassica rapa***

Observe the *Brassica rapa* leaves from each treatment group using LSCM, with an excitation wavelength of 540 nm and an emission wavelength of 580 nm.

**Text S4.5.2 The absorption and transport of CDs in *Brassica rapa***

Observing the absorption and transport of CDs in *Brassica rapa* leaves across different treatment groups using LSCM, with an excitation wavelength of 360 nm and an emission wavelength of 436 nm.

**Text S4.6. Measurement of reactive oxygen species content and cell membrane integrity**

Following the method of Li et al. [4], ROS levels and cell membrane integrity in *Brassica rapa* leaves were assessed using histochemical staining. The staining solutions employed included DAB solution (0.05 g/45 mL, pH 3.8), NBT solution (0.1 g/50 mL, sodium phosphate buffer), and 0.25% Evans Blue solution. Leaf samples were collected on days 35 and 45. After incubation overnight at room temperature in the dark with the respective staining solutions and subsequent washing, decolorization was performed using a glycerol: ethanol (1:9) mixture in a boiling water bath. Finally, the stained areas and intensities were quantified using ImageJ software.

**Text S5.** **Characterization of polyethylene NPs (PE) and tire wear particles (TWP) and their enrichment on the surface of plant leaves.**

**Text S5.1. Characterization of PE and TWP.**

The morphological features of PE and TWP were observed using SEM, and the particle size range of PE was calculated using Image J.

**Text S5.2. The enrichment of PE and TWP on plant leaves.**

The *Brassica rapa* seedlings with consistent growth were selected for the experiment. For the two types of microplastics, PE and TWP, four treatment groups were set up respectively: the control group (CK), the group treated with CS-CDs alone, the group treated with microplastics alone (PE or TWP), and the group treated with the combination of microplastics and CS-CDs (PE+CS-CDs or TWP+CS-CDs). The application method of microplastics followed the method of Wang et al. [1] , and the application dose was consistent with the PS group, 150 μg per plant per day. The composite treatment group sprayed CS-CDs evenly on the leaf surface once before the first application of microplastics; the CS-CDs alone treatment group sprayed in the same way. The CK group sprayed the same amount of deionized water. The treatment period was 10 days. After the treatment, the *Brassica rapa* leaves under different conditions were collected for freeze-drying. Then, the leaf surfaces were observed under a SEM in a high-vacuum mode to explore the aggregation state of PE and TWP around the stomata and their surrounding areas under different treatments.

**References**

[1] Y. Wang, L. Xiang, F. Wang, Z. Wang, Y. Bian, C. Gu, X. Wen, F.O. Kengara, A. Schäffer, X. Jiang, B. Xing, Positively Charged Microplastics Induce Strong Lettuce Stress Responses from Physiological, Transcriptomic, and Metabolomic Perspectives, Environmental Science & Technology, 56 (2022) 16907-16918.Http//dx.doi.org/10.1021/acs.est.2c06054

[2] F. Khodadadi, F.S. Ahmadi, M. Talebi, N. Moshtaghi, A. Matkowski, A. Szumny, M. Rahimmalek, Essential oil composition, physiological and morphological variation in Salvia abrotanoides and S. yangii under drought stress and chitosan treatments, Industrial Crops and Products, 187 (2022).Http//dx.doi.org/10.1016/j.indcrop.2022.115429

[3] Y. Liu, D. Liu, X. Han, Z. Chen, M. Li, L. Jiang, J. Zeng, Magnesium-Doped Carbon Quantum Dot Nanomaterials Alleviate Salt Stress in Rice by Scavenging Reactive Oxygen Species to Increase Photosynthesis, ACS Nano, 18 (2024) 31188-31203.Http//dx.doi.org/10.1021/acsnano.4c09001

[4] M. Li, B. Zhao, X. Han, J. Jiang, C. Liu, Y. Liu, Bioplastics (PC/GL) versus non-biodegradable plastics (PE/PP): Effects on growth and rhizosphere soil microbial-metabolomics of Houttuynia cordata, Journal of Hazardous Materials, 498 (2025).Http//dx.doi.org/10.1016/j.jhazmat.2025.139794

**Figure S1****.** Size distribution of CDs.





**Figure S2.** (A) Total chlorophyll content. (B) Carotenoid content. FW: Fresh weight. Data are presented as mean ± standard deviation (n = 6). Bars indicated with different letters indicate significantly different values (*p*< 0.05).


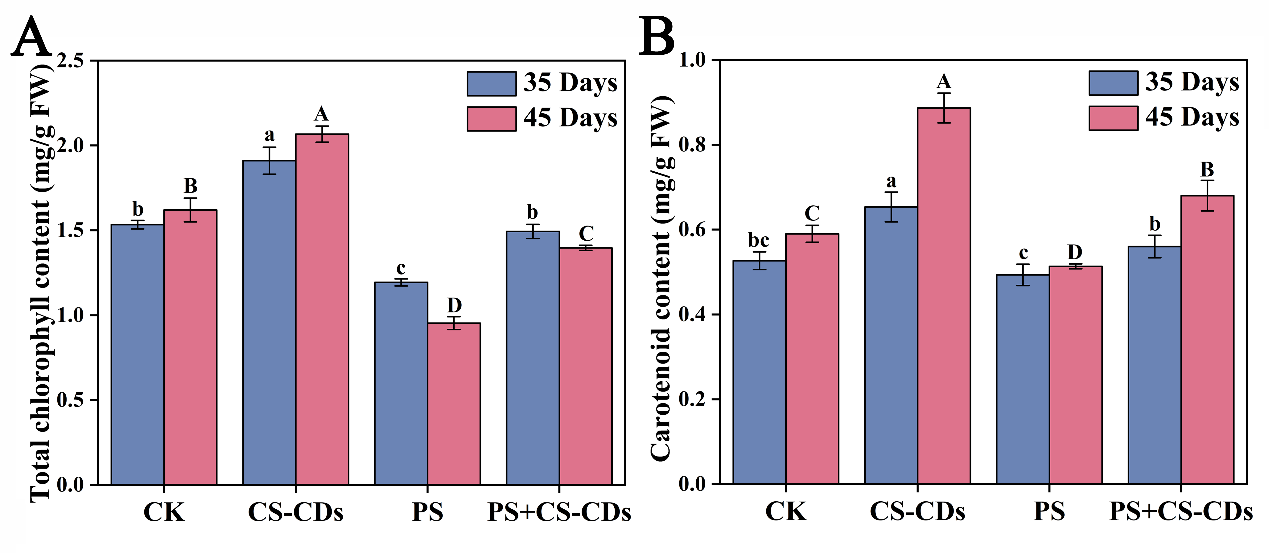


**Figure S3.** (A) MDA content. (B) POD activity. (C) SOD activity. (D) CAT activity. FW: Fresh weight. Data are presented as mean ± standard deviation (n = 6). Bars indicated with different letters indicate significantly different values (*p* < 0.05).


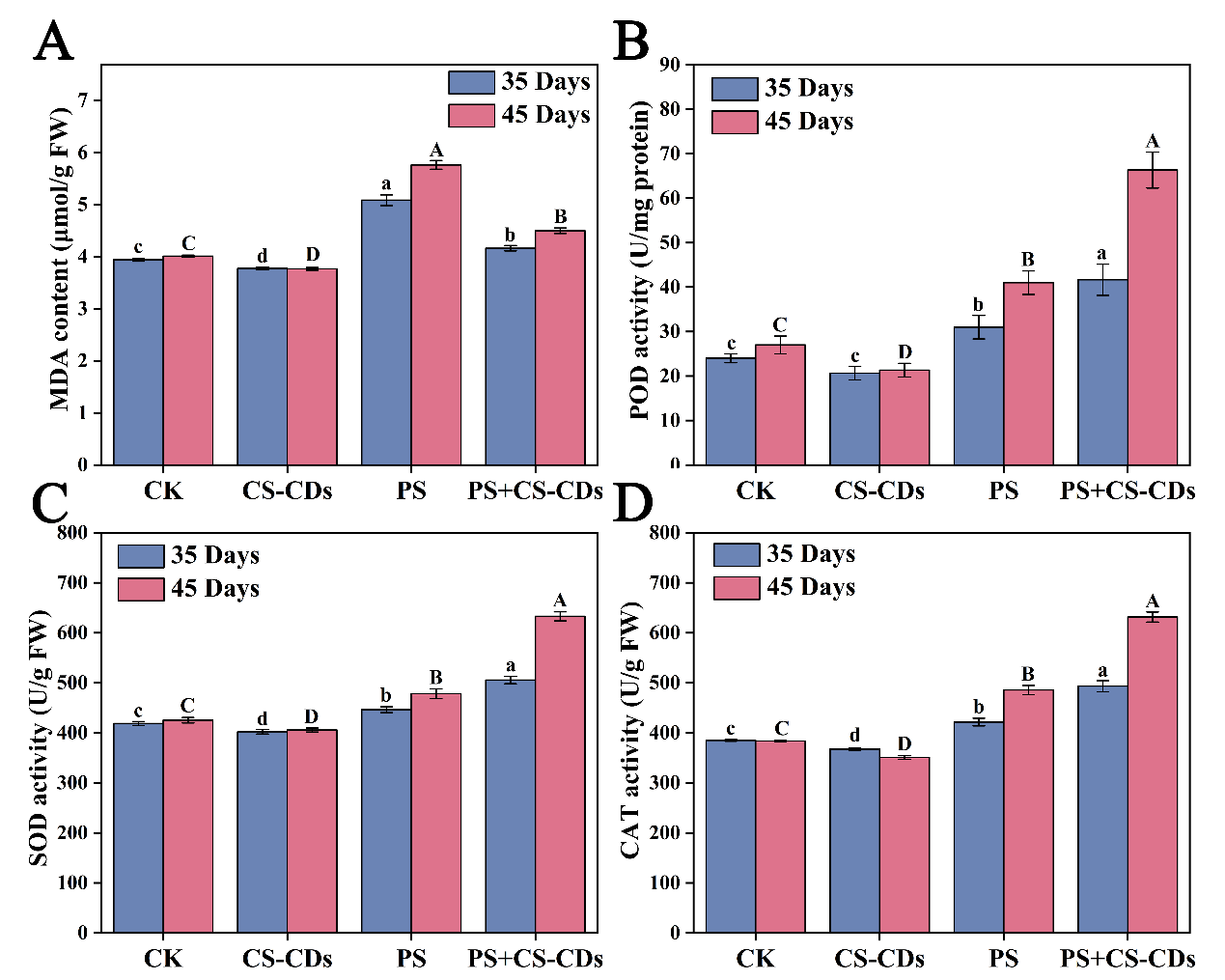


**Figure S4.** The distribution of stomata and surrounding PE in the leaves of Brassica rapa in different treatment groups. Scale bar = 5.00 μm/1.00 μm.

**
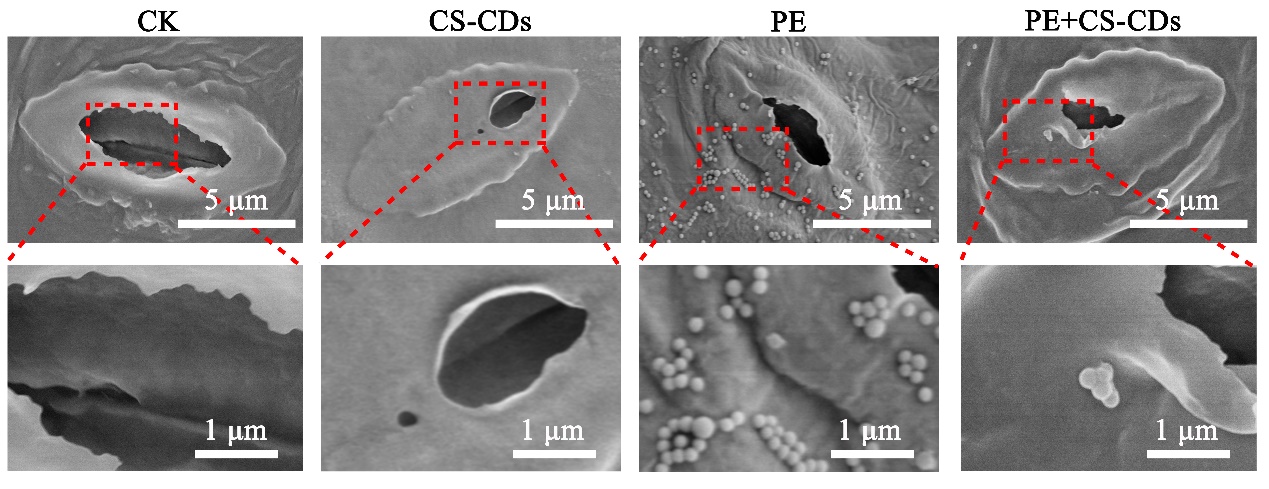
**

**Figure S5.** The enrichment of TWP in the stomata and surrounding areas of *Brassica rapa* leaves in different treatment groups. Scale bar = 5.00 μm/1.00 μm.

**
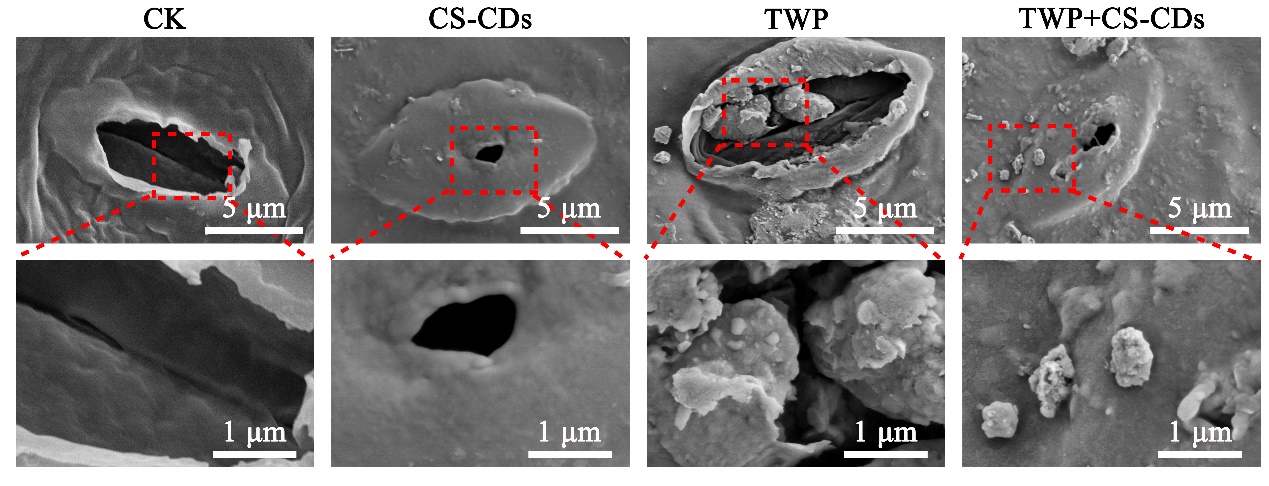
**

**Figure S6.** Different treatment groups had different effects on the enrichment pathways of metabolites in *Brassica rapa* leaves. (A) The effect of CS-CDs on the metabolite enrichment pathway of *Brassica rapa* leaves compared with CK. (B) The effect of PS on the enrichment pathway of metabolites in *Brassica rapa* leaves compared with CK. (C) The effect of PS+CS-CDs on the enrichment pathway of metabolites in *Brassica rapa* leaves compared with CK. (D) The effect of PS+CS-CDs on the enrichment pathway of metabolites in *Brassica rapa* leaves compared with PS.

**
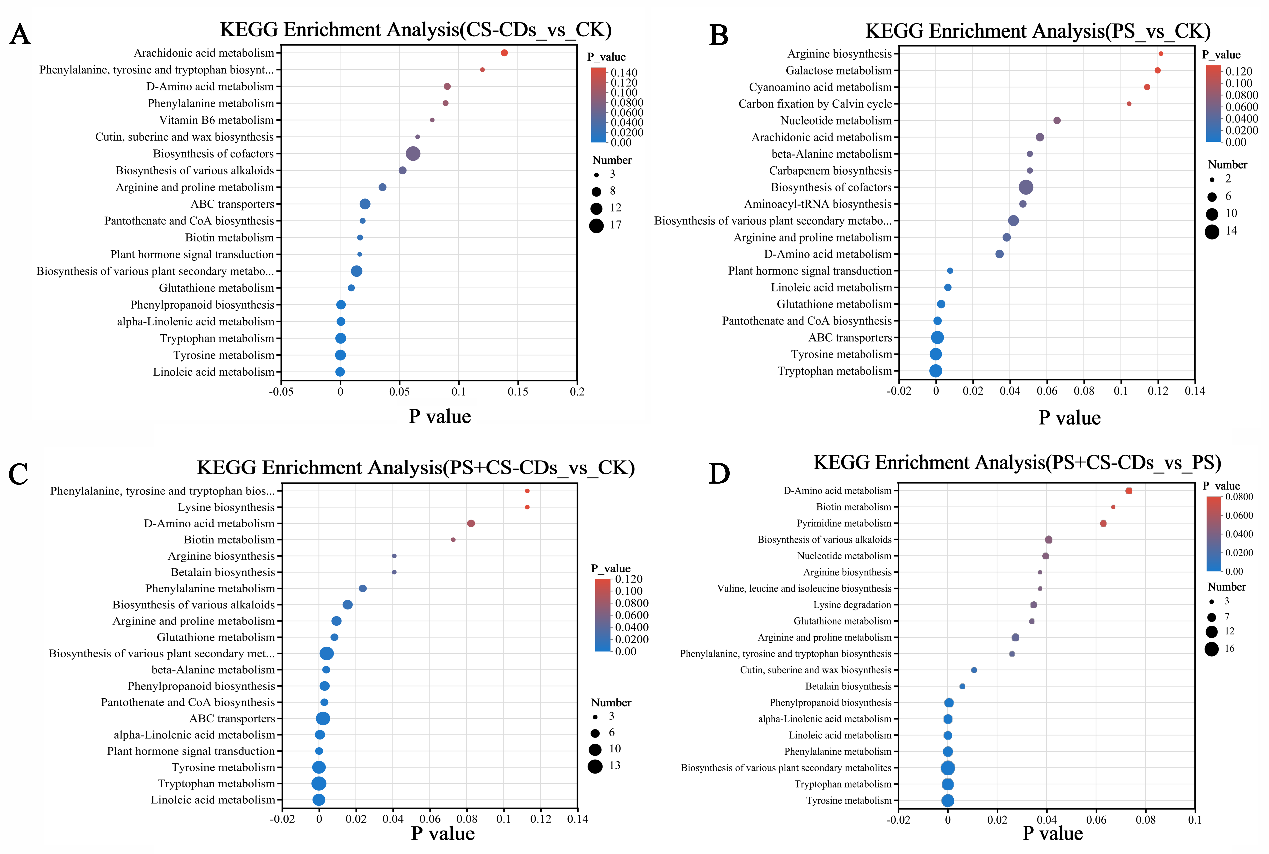
**

**Figure S7.** (A) SEM image of PS. Scale bar = 1.00 μm. (B) Size distribution of PS.


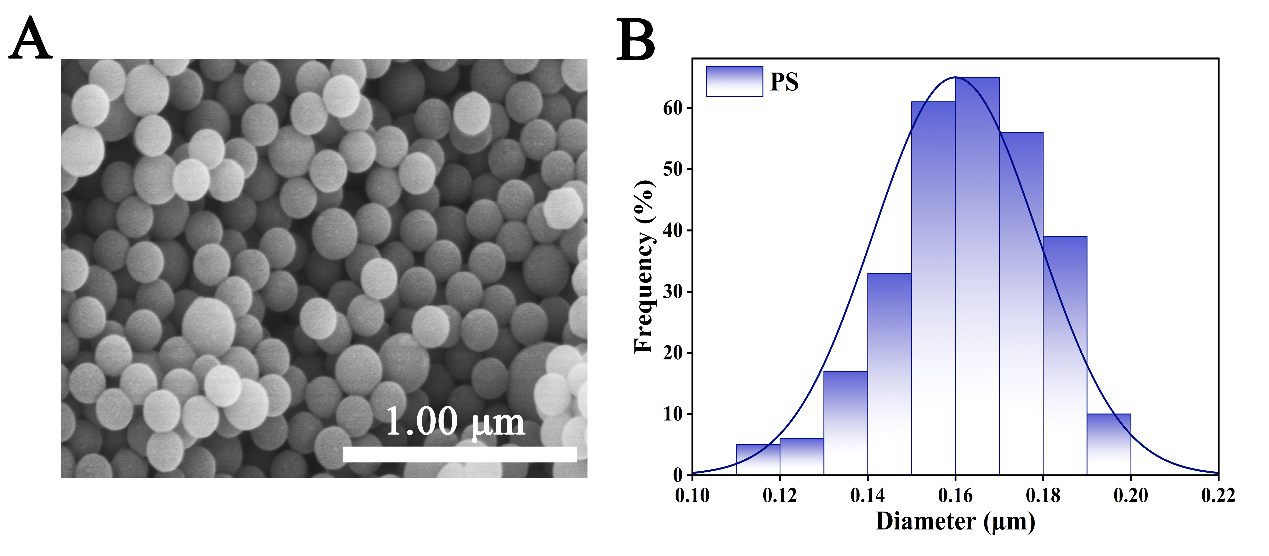


**Figure S8.** (A) SEM image of PE. Scale bar = 1.00 μm. (B) Size distribution of PE.


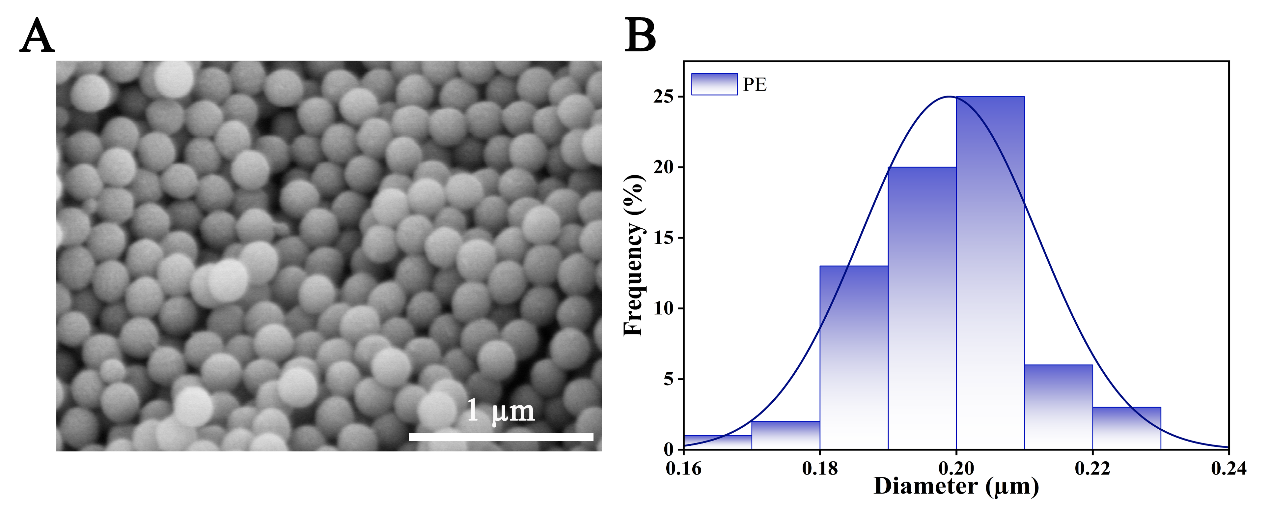


**Figure S9.** SEM image of TWP. Scale bar = 100.00 μm.


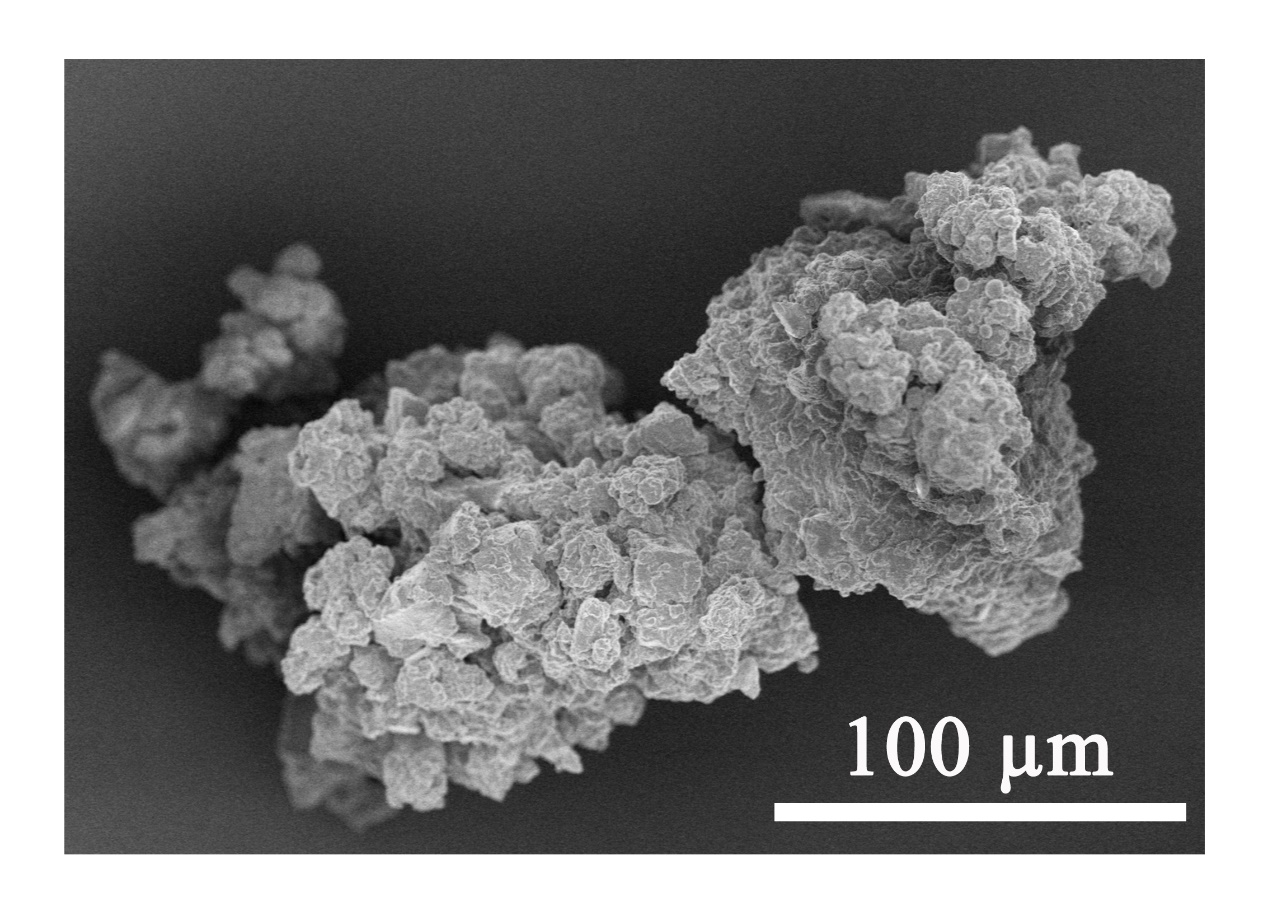


**Figure S10.** PS enrichment around the stomata of *Brassica rapa* plants was observed using SEM and LSCM. (A) SEM images showing PS enrichment around the stomata at different concentrations. Scale bar = 5 μm/1 μm. (B) LSCM images showing PS accumulation in the leaves at different concentrations. Scale bar = 10 μm.


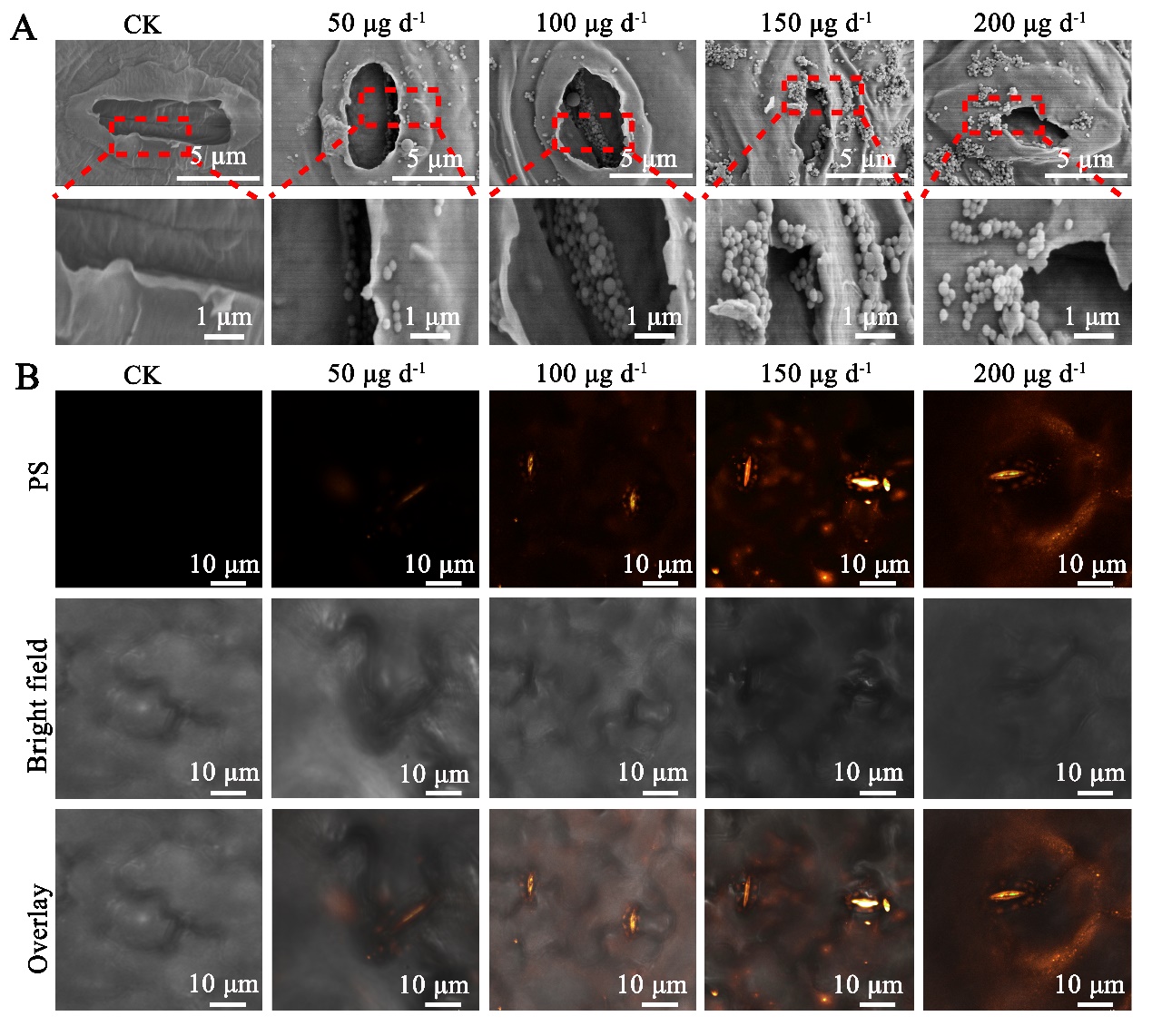


**Figure S11.** The enrichment of different concentrations of CS-CDs in the stomata and surrounding areas of the *Brassica rapa* leaves was observed using SEM, as well as the cross-sectional and surface morphology of the self-forming films. (A) The adhesion of CK and different concentrations of CS-CDs composite solutions on the leaves of *Brassica rapa*. Scale bar = 10 μm/50 μm. (B) The cross-sectional morphology of the independent films formed by different concentrations of CS-CDs composite solutions. Scale bar = 100 μm. (C) The surface structure morphology of the independent films formed by different concentrations of CS-CDs composite solutions. Scale bar = 100 μm.


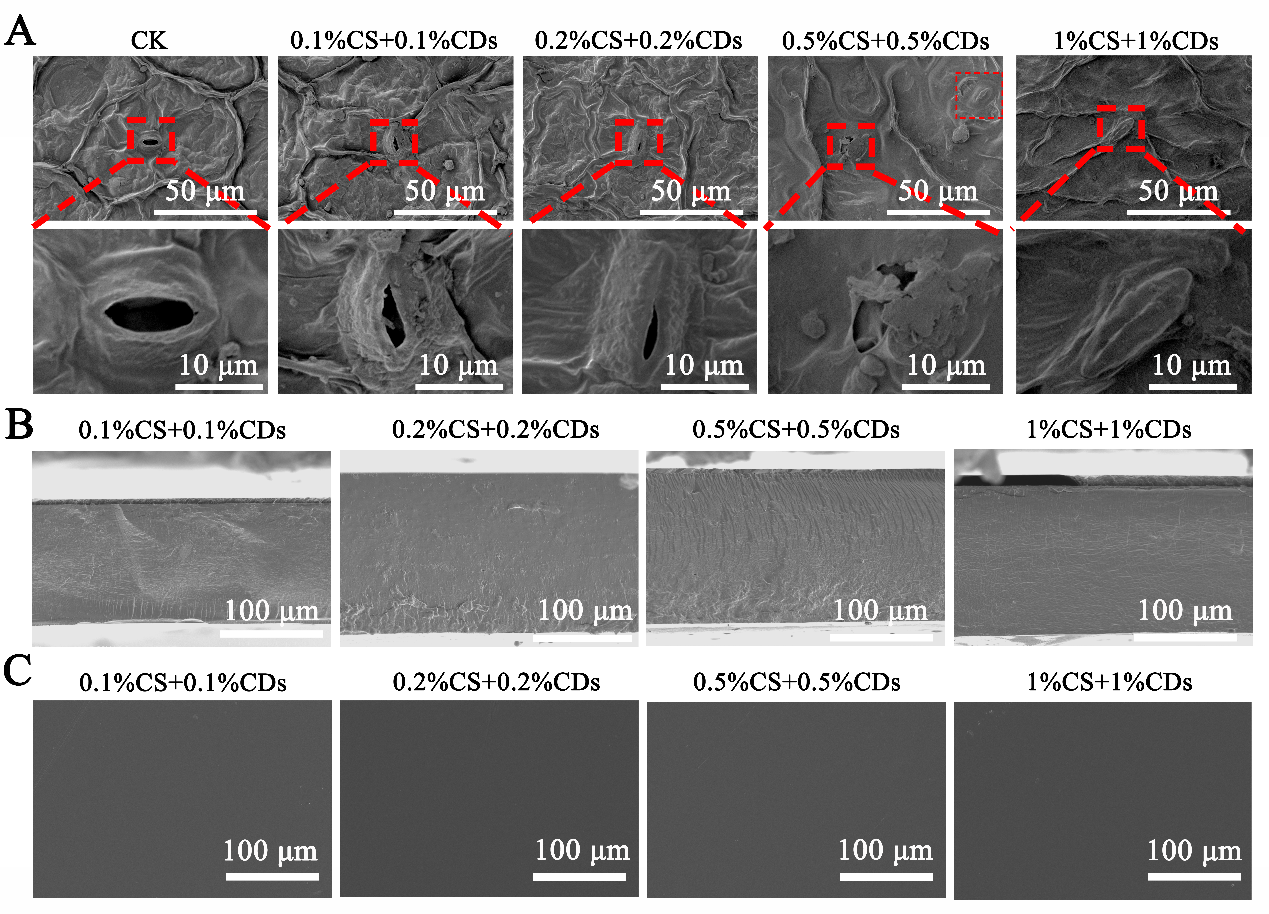

Supplement: Supplementary file 1 — Supporting File: advs75278‐sup‐0001‐SuppMat.docx. [file ADVS-13-e75278-s001.docx]
